# Engineering of *Corynebacterium glutamicum* for biosynthesis of the pharmaceutically active *N*-acetyltyramine: establishing and optimizing *de novo* production

**DOI:** 10.1186/s13036-026-00713-1

**Published:** 2026-06-18

**Authors:** Sara-Sophie Poethe, Lilli Sophie Kaimann, Kai H. Schülke, Stephan C. Hammer, Volker F. Wendisch

**Affiliations:** 1 Genetics of Prokaryotes, Faculty of Biology and CeBiTec, Bielefeld University, Universitätsstr. 25, 33615 Bielefeld, Germany; 2 Organic Chemistry and Biocatalysis, Faculty of Chemistry and CeBiTec, Bielefeld University, Universitätsstr. 25, 33615 Bielefeld, Germany

**Keywords:** *Corynebacterium glutamicum*, *N*-Acetyltyramine, *N*-Propionyltyramine, Arylalkylamine *N*-acetyltransferase, Design of experiments

## Abstract

**Background:**

*N*-Acetyltyramine is increasingly recognized for its diverse pharmaceutically relevant properties. In particular, it exhibits antimicrobial activity against multi-drug resistant pathogens, which pose a considerable health challenge. Therefore, the demand for a sustainable and efficient *N*-acetyltyramine production arises.

**Results:**

Here, we engineered *Corynebacterium glutamicum* for *de novo* production of *N*-acetyltyramine. Heterologous overexpression of different bacterial and insect acetyltransferase genes in a tyramine overproducing strain identified the arylalkylamine *N*-acetyltransferase from *Bombyx mori* as the most promising enzyme, resulting in an *N*-acetyltyramine titer of 5.5 ± 0.3 mM. Design of Experiment-based optimization of the culture medium revealed urea and l-phenylalanine as media components significantly affecting *N*-acetyltyramine production. Combining media optimization with genetic engineering to balance the expression strength of the genes encoding the l-tyrosine decarboxylase from *Levilactobacillus brevis* and the arylalkylamine *N*-acetyltransferase from *B. mori* increased *de novo N*-acetyltyramine production to 13.8 ± 0.1 mM (2.5 ± 0.1 g/L). The optimized medium composition was also transferable to the production of other l-tyrosine derivatives, tyramine and tyrosol, increasing their titers by 24% and 44%, respectively. In addition, supplementing the established *N*-acetyltyramine producing strain with propionate as a second carbon source enabled the production of *N*-propionyltyramine. To the best of our knowledge, this represents the first report describing biotechnological production of *N*-propionyltyramine.

**Conclusion:**

In this study, *de novo N*-acetyltyramine production was established and substantially increased through the combined implementation of metabolic engineering and media optimization strategies. The successful transfer of the optimized medium to enhance the production of additional l-tyrosine derivatives underscores the potential of integrating genetic engineering with culture media refinement to improve biotechnological processes.

**Supplementary Information:**

The online version contains supplementary material available at 10.1186/s13036-026-00713-1.

## Introduction

The increasing number of multidrug-resistant pathogenic bacteria represents a major global health threat and is one of the most pressing medical challenges of our time [1, 2]. *N*-Acetyltyramine (*N*-(4-hydroxyphenethyl)acetamide) has attracted growing attention as a promising antibacterial compound for the treatment of antibiotic-resistant infections, as it has been described to be active against a variety of multidrug-resistant pathogens, including *Staphylococcus aureus*,* Escherichia coli*, and *Pseudomonas aeruginosa* [3]. While the mechanism of inhibition is undetermined, *N*-acetyltyramine reduces both pyocyanin production and biofilm formation [4]. Its minimal inhibitory concentration (MIC) of 10–30 µg mL^− 1^ [3] is similar to the MICs of antibiotics currently used to combat these pathogens, such as vancomycin or the ceftolozane-tazobactam combination [5, 6]. Besides its antimicrobial potential, *N*-acetyltyramine possesses multiple further pharmaceutically relevant properties as it restores the sensitivity of cancer cells to the chemotherapeutic agent doxorubicin [7], is a radical-scavenging antioxidant [8], and acts anti-adipogenic by inhibiting lipid accumulation and the differentiation of adipocytes [9]. Moreover, it inhibits the coagulation factor Xa and platelet aggregation, suggesting its potential as an antithrombotic compound for the treatment of cardiovascular diseases [10].

The growing pharmaceutical interest in *N*-acetyltyramine has created a demand for its efficient and sustainable production. In nature, *N*-acetyltyramine is produced by various microorganisms, including actinobacteria [8, 9, 11, 12], as well as the *Tenebrio molitor* larvae [10]. Metabolic engineering employing acetyltransferases for the enzymatic *N*-acetylation of tyramine enables the biotechnological production of *N*-acetyltyramine from simple carbon and nitrogen sources.

**Fig. 1:**
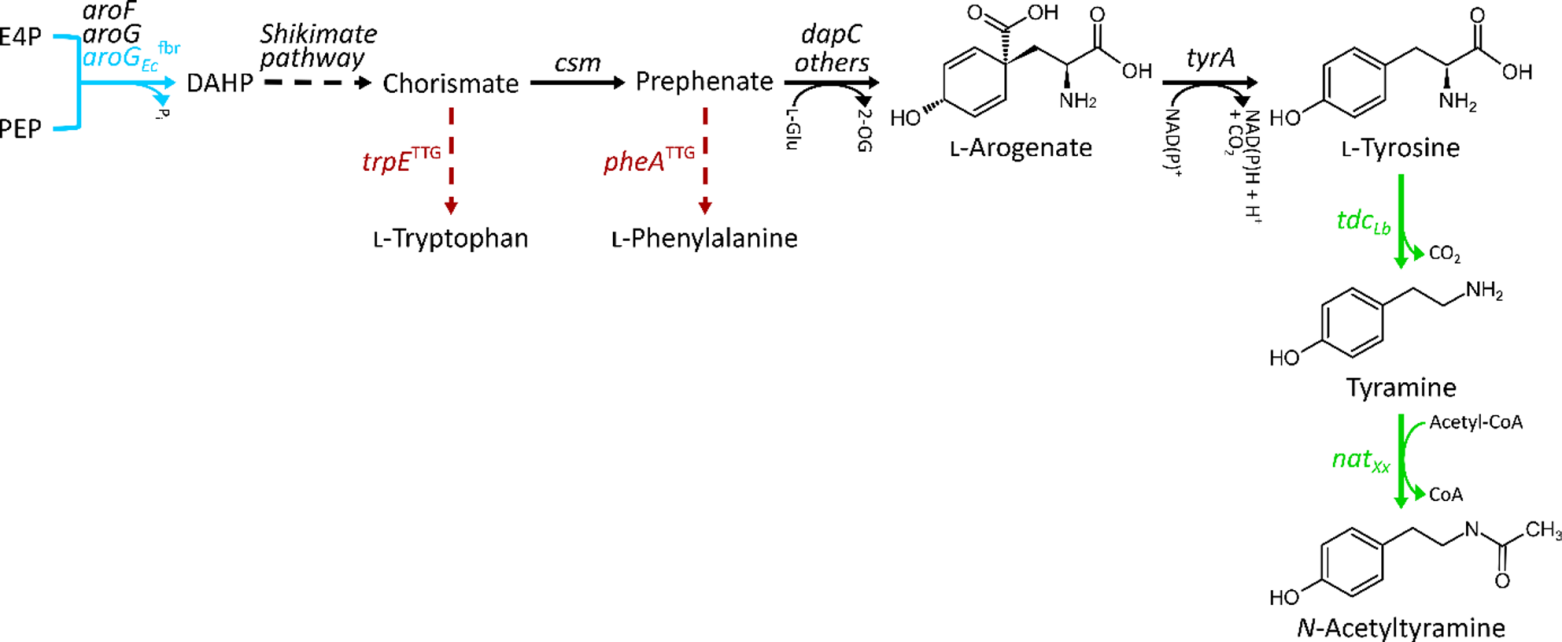
Metabolic engineering of *C. glutamicum* for the biosynthesis of *N*-acetyltyramine. Genetic engineering includes the chromosomal integration of *aroG*_*Ec*_^fbr^ (blue), reduced expression of *trpE* and *pheA* by start codon exchange from ATG to TTG (red), and plasmid-based overexpression of *tdc*_*Lb*_ and an *N*-acetyltransferase gene (*nat*, green). Dashed arrows indicate multiple reaction steps. Genes encoding the enzymes catalyzing the corresponding reactions include: *aroF* and *aroG*: DAHP synthase, *aroG*_*Ec*_^fbr^: feedback-inhibition resistant DAHP synthase mutant from *E. coli*, *csm*: chorismate mutase, *dapC*: *N*-succinyl-diaminopimelate aminotransferase, *nat*_*Xx*_: *N*-acetyltransferase from different organisms, *pheA*: prephenate dehydratase, *trpE*: anthranilate synthase, *tyrA*: l-arogenate dehydrogenase, *tdc*_*Lb*_: tyrosine decarboxylase from *Levilactobacillus brevis*. Abbreviations: 2-OG: 2-oxoglutarate, CoA: coenzyme A, DAHP: 3-deoxy-d-arabinoheptosulosonate-7-phosphate, E4P: erythrose 4-phosphate, l-Glu: l-glutamate, NAD(P): nicotinamide adenine dinucleotide (phosphate), PEP: phosphoenolpyruvate, P_i_: inorganic phosphate

*Corynebacterium glutamicum* has been engineered for the production of numerous compounds generally recognized as safe (GRAS) for the food, feed, and health industry [13]. These include terpenes such as *trans*-nerolidol [14] or astaxanthin [15], amino acids [16, 17], including a variety of aromatic compounds [18, 19], as well as antimicrobial agents such as roseoflavin [20] or the antimicrobial peptides pediocin PA-1 [21] and garvicin Q [22]. Previous metabolic engineering resulted in a *C. glutamicum* strain overproducing tyramine in the gram-per-liter scale [23], rendering it a promising candidate for *N*-acetyltyramine biosynthesis (Fig. 1). To ensure a high l-tyrosine precursor supply, a feedback-inhibition resistant mutant of the *E. coli* 3-deoxy-d-arabinoheptosulosonate-7-phosphate (DAHP) synthase gene (*aroG*_*Ec*_^fbr^) has been integrated into its genome. To reduce competing l-tryptophan and l-phenylalanine biosynthesis, the translational start codons of the genes encoding the anthranilate synthase (*trpE*) and the prephenate dehydratase (*pheA*) were changed from ATG to the less frequently used TTG [24]. Upon plasmid-based overexpression of the tyrosine decarboxylase gene from *Levilactobacillus brevis* (*tdc*_*Lb*_), a tyramine titer of 1.9 g L^− 1^ was achieved with the resulting strain AROM3 (*tdc*_*Lb*_) [23], hereinafter referred to as strain TRN. Overexpression of an *N*-specific acetyltransferase gene in strain TRN is expected to extend its tyramine biosynthesis pathway toward *N*-acetyltyramine production (Fig. 1).

Complementing metabolic engineering, media optimization is increasingly recognized for its potential to enhance biotechnological processes [25]. Adjusting the composition of the culture medium can positively affect metabolic processes by influencing cellular physiology. For example, the stepwise optimization of the concentrations of the carbon, nitrogen, and phosphate sources in the cultivation medium increased shikimic acid production by *Bacillus licheniformis* [26]. However, while optimizing only one factor at a time is common and simple, this approach is costly and time-consuming, and does not consider interactions between different factors [27]. These limitations can be partially addressed by incorporating machine learning to predict and evaluate optimal medium compositions based on data sets comprising various medium compositions for production. Such strategies have been successfully applied to enhance 4-aminophenylalanine production and more than doubled l-tyrosine production titers by *E. coli* [28], as well as increased flaviolin titers produced by *Pseudomonas putida* by 70% through media optimization [29]. However, these approaches necessitate high-throughput screening of numerous media compositions to generate sufficient training data for the algorithms. This makes them expensive and often requires (semi-)automated cultivation platforms. In contrast, statistical methods, such as the Design of Experiments (DoE) approach, enable to systematically test multiple parameters and their interactions simultaneously, thereby reducing the number of experiments while still allowing the identification and optimization of relevant medium components and their interactions [27, 30]. DoE has been successfully employed to optimize *C. glutamicum* cultivation media for the production of carotenoids [15], terpenes [14], amino acids [31], muconic acid [32], the heterocyclic aromatic 2,3,5,6-tetramethylpyrazine [33], and the shikimate pathway derived protocatechuic acid (PCA) [34].

In this study, metabolic engineering was combined with DoE-based media optimization to enable and optimize *de novo* production of *N*-acetyltyramine. Bacterial and insect acetyltransferases were assessed for the acetylation of tyramine. The heterologous gene expression was balanced, and the composition of the cultivation medium was refined to increase *N*-acetyltyramine titers. Additionally, propionate supplementation was assessed for its potential to broaden the product spectrum.

## Materials and methods

### Bacterial strains and cultivation conditions

Bacterial strains and plasmids used in this study are listed in Tables 1 and 2, respectively. *E. coli* DH5α served as a cloning host and was cultivated in lysogeny broth (LB) medium [35] at 37 °C and 180 rpm.

For production experiments, *C. glutamicum* was first cultivated in 100 mL baffled shake flasks containing 10 mL LB medium supplemented with 10 g L^− 1^ glucose at 30 °C and 120 rpm for 8 h. This first preculture was then used to inoculate 50 mL CGXII minimal medium [36] to an initial optical density at 600 nm (OD_600_) of 1 as a second preculture. After overnight cultivation, the main culture was inoculated to an OD_600_ of 1. To assess production of *N*-propionyltyramine, a preculture of strain ATRN in 10 mL LB medium containing 10 g L^− 1^ sodium-propionate cultivated at 30 °C and 120 rpm for 8 h was used for inoculation of the main culture. Unless stated otherwise, production experiments were performed in the BioLector XT microcultivation system 48-well Flower Plates with a filling volume of 1 mL (Beckmann Coulter, Brea, CA, United States) at 1,100 rpm for 120 h, and CGXII minimal medium was supplemented with 40 g L^− 1^ glucose and 0.5 mM l-phenylalanine, as well as 1 mM isopropyl-β-d-1-thiogalactopyranoside (IPTG) to induce plasmid-based gene expression. Where appropriate, 25 mg L^− 1^ kanamycin, 5 mg L^− 1^ tetracycline, or 100 mg L^− 1^ spectinomycin was added.

To assess potential toxic effects and degradation of *N*-acetyltyramine, cultivations were performed in 100 mL baffled shake flasks with a filling volume of 10% (v v^− 1^) in CGXII minimal medium, additionally containing up to 20 mM *N*-acetyltyramine for 48 h. Growth was monitored with a V-1200 spectrophotometer (VWR, Radnor, PA, USA). The measured OD_600_ was converted into cell dry weight concentration (CDW) using an experimentally determined correlation factor: CDW [g L^− 1^] = OD_600_ × 0.347 [37].

**Table 1:** Bacterial strains used in this study

Strain	Relevant characteristics	Antibiotic resistance	Supplements	Reference
***E. coli***				
DH5α	*supE44* Δ*lacU169* (Φ*80lacZ* Δ*M15*) *hsdR17 recA1 endA1 gyrA96 thi-1 relA1*	-	-	[38]
DH5α (AANAT_*Ae*_)	DH5α carrying (pVWEx4-AANAT_*Ae*_)	Kan^R^	-	This study
DH5α (pSJEx3-AANAT_*Bm*_)	DH5α carrying (pSJEx3-AANAT_*Bm*_)	Spec^R^	-	This study
DH5α (pVWEx1-AANAT_*Bm*_)	DH5α carrying (pVWEx1-AANAT_*Bm*_)	Kan^R^	-	This study
DH5α (AANAT_*Bm*_)	DH5α carrying (pVWEx4-AANAT_*Bm*_)	Kan^R^	-	This study
DH5α (AcT_*Ms*_)	DH5α carrying (pVWEx4-AcT_*Ms*_)	Kan^R^	-	This study
DH5α (NAT1_*Mj*_)	DH5α carrying (pVWEx4-NAT1_*Mj*_)	Kan^R^	-	This study
***C. glutamicum***				
AROM3	C1* ∆*ldhA* ∆*vdh*::P_*ilvC*_-*aroG*_*Ec*_^D146N^*trpE*^TTG^*pheA*^TTG^	-	l-Phenylalanine	[24]
TRN	AROM3 carrying (pECXT99A-*tdc*_*Lb*_)	Tet^R^	l-Phenylalanine	[23]
TRN (EV)	TRN carrying (pVWEx4)	Tet^R^, Kan^R^	l-Phenylalanine	[37]
TRN (pVWEx4-AANAT_*Ae*_)	TRN carrying (pVWEx4-AANAT_*Ae*_)	Tet^R^, Kan^R^	l-Phenylalanine	This study
TRN (pSJEx3-AANAT_*Bm*_)	TRN carrying (pSJEx3-AANAT_*Bm*_)	Tet^R^, Spec^R^	l-Phenylalanine	This study
ATRN	TRN carrying (pVWEx1-AANAT_*Bm*_)	Tet^R^, Kan^R^	l-Phenylalanine	This study
TRN (pVWEx4-AANAT_*Bm*_)	TRN carrying (pVWEx4-AANAT_*Bm*_)	Tet^R^, Kan^R^	l-Phenylalanine	This study
TRN (pVWEx4-AcT_*Ms*_)	TRN carrying (pVWEx4-AcT_*Ms*_)	Tet^R^, Kan^R^	l-Phenylalanine	This study
TRN (pVWEx4-NAT1_*Mj*_)	TRN carrying (pVWEx4-NAT1_*Mj*_)	Tet^R^, Kan^R^	l-Phenylalanine	This study
TRN (pVWEx4-*tyo*_*Kr*_)	TRN carrying (pVWEx4-*tyo*_*Kr*_)	Tet^R^, Kan^R^	l-Phenylalanine	[37]

**Table 2:** Plasmids used in this study

Plasmid	Relevant characteristics	Antibiotic resistance	Reference
pSJEx3	*C. glutamicum*/*E. coli* shuttle vector for inducible overexpression, P_*tac*_, *lacI*^q^, pBL1 oriV_*Cg*_	Spec^R^	[39]
pSJEx3-AANAT_*Bm*_	Derivative of pSJEx3 for overexpression of the codon-harmonized gene encoding AANAT from *B. mori* with an optimized RBS	Spec^R^	This study
pVWEx1	*C. glutamicum*/*E. coli* shuttle vector for inducible overexpression, P_*tac*_, *lacI*^q^, pHM1519 oriV_*Cg*_	Kan^R^	[40]
pVWEx1-AANAT_*Bm*_	Derivative of pVWEx1 for overexpression of the codon-harmonized gene encoding AANAT from *B. mori* with an optimized RBS	Kan^R^	This study
pVWEx4	pVWEx1 derivative with mutation *repA*^G429E^	Kan^R^	[41]
pVWEx4-AANAT_*Ae*_	Derivative of pVWEx4 for overexpression of the codon-harmonized gene encoding AANAT from *Acromyrmex echinatior* with an optimized RBS	Kan^R^	This study
pVWEx4-AANAT_*Bm*_	Derivative of pVWEx4 for overexpression of the codon-harmonized gene encoding AANAT from *B. mori* with an optimized RBS	Kan^R^	This study
pVWEx4-AcT_*Ms*_	Derivative of pVWEx4 for overexpression of the gene encoding AcT from *Mycobacterium smegmatis* with an optimized RBS	Kan^R^	This study
pVWEx4-NAT1_*Mj*_	Derivative of pVWEx4 for overexpression of the codon-harmonized gene encoding NAT1 from *Mesorhizobium japonicum* with an optimized RBS	Kan^R^	This study

Gene sequences of codon-harmonized genes used for plasmid construction in this study are listed in Supplementary Table S1. Plasmids for the overexpression of genes encoding acetyltransferases are named after the enzyme they encode

### Molecular biological methods

PCR, plasmid isolation, and DNA purification were performed as previously described [23]. Sequences of the genes encoding AANAT_*Ae*_, AANAT_*Bm*_, and NAT1_*Mj*_ were codon-harmonized for *C. glutamicum* [42], and an optimized ribosome binding site (RBS) was calculated for each gene [43] (Supplementary Table S1). Synthesized gene fragments were purchased from Twist Bioscience (San Francisco, CA, United States), vector-specific overhangs were added with primers (Supplementary Table S2), and the genes were cloned into BamHI-restricted plasmids (Thermo Fisher Scientific, Waltham, MA, United States) via Gibson assembly [44]. The resulting Gibson assembly mixture was used to transform chemocompetent *E. coli* DH5α [45], and the cloned plasmids were verified by sequencing. *N*-Acetyltyramine producing strains were constructed by transforming electrocompetent *C. glutamicum* TRN via electroporation followed by a heat shock at 46 °C [36].

### Design of experiments

Experiments for media optimization were designed and analyzed with R 4.4.1 [46] using the rsm package version 2.10.5 [47]. For all experiments, the standard CGXII minimal medium composition served as center point (defined as 100%). Cube portions corresponded to combinations of 50% and 150% of the standard concentrations of the components according to the corresponding design. For circumscribed star points, the concentration of one component was set to 25% or 175% of its standard concentration, while all other components were kept constant at center point level. Stock solutions for adjusting the various media compositions were prepared as previously described [15], and cultivations were performed in the BioLector system as described before.

To identify candidate media components affecting *N*-acetyltyramine production, a 2^15−10^ fractional factorial design was employed as a screening model, using different combinations of the effects of the macronutrients ammonium sulfate, urea, phosphate, MOPS, and glucose for confounding the remaining ten factors, as described in “Experiments: Planning, Analysis and Optimization” [48]. The screening design was divided into two blocks of BioLector cultivations: The first block comprised the cube portions and four center points, and the second block included the circumscribed star points and three center points (Supplementary Table S3).

For the response surface methodology (RSM) experiment, a 2^6−1^ fractional factorial design was employed, including four center points, the cube portions, and the circumscribed star points. For the cube portions, the effect of l-phenylalanine was confounded with the combined effects of urea, CaCl_2_, FeSO_4_, MnSO_4_, and biotin (Supplementary Table S4). Subsequently, a steepest ascent analysis was performed using the results of the RSM. The first five conditions along the path of steepest ascent (distances 0.5–2.5; Supplementary Table S5), along with a center point, were evaluated in triplicates.

### Activity assay of acetyltransferases

For protein overproduction, 500 mL LB medium was inoculated with *E. coli* DH5α strains carrying the pVWEx4 plasmid for overexpression of the respective acetyltransferase gene, or the empty vector control, to an initial OD_600_ of 0.05. At an OD_600_ of 0.5, gene expression was induced by adding 0.5 mM IPTG. Cells were cultivated at 21 °C and 180 rpm for 5 h, harvested by centrifugation, and stored at -20 °C. Frozen cell pellets were resuspended in TNG buffer (20 mM Tris-HCl, 300 mM NaCl, 5% (w v^− 1^) glycerol) containing 1 mM phenylmethylsulfonyl fluoride (PMSF), and cell lysis was performed by sonication (UP200S, Hielscher Ultrasonics GmbH, Teltow, Germany). Lysates were centrifuged at 4 °C and 14,000 rpm for 60 min, and the supernatants were purified using PD-10 desalting columns (Cytiva, Marlborough, MA, USA), eluting the crude extract with the phosphate buffer used in the respective enzyme assay. Protein concentrations of the crude extracts were determined using the Bradford assay with bovine serum albumin as standard [49].

To screen bacterial and insect acetyltransferases for *N*-acetylation of tyramine, enzyme assays were performed in 0.1 M phosphate buffer (pH 8.0) containing 25 mM tyramine and 0.5 mM acetyl-CoA. Reactions were started by addition of crude extract, using three different crude extract concentrations for each enzyme. The apparent kinetics of AANAT_*Bm*_ for acetyl-CoA and propionyl-CoA were determined in enzyme assays with 10 mM tyramine and 50 µg mL^− 1^ crude extract in 0.1 M phosphate buffer (pH 7.5), using varying concentrations of the respective acyl-CoA. All enzyme assays were performed at 30 °C. Samples were taken in regular intervals, reactions were stopped by mixing the sample with an equal volume of methanol, and samples were stored at -20 °C until HPLC analysis.

To determine apparent *V*_max_, *K*_M_, and *K*_I_ values, the initial velocities *v*_*o*_ measured for the tested acetyl-CoA and propionyl-CoA concentrations were fitted to the Michaelis-Menten equation and Haldane equation, respectively, using Excel Solver (Microsoft, Redmond, WA, USA), minimizing the sum of squared errors.

### Quantification of aromatic compounds and carbohydrates by HPLC analysis

Extracellular levels of l-tyrosine, tyramine, *N*-acetyltyramine, and *N*-propionyltyramine were quantified using a high-performance liquid chromatography system (1200 series, Agilent Technologies Deutschland GmbH, Waldbronn, Germany). Culture supernatants taken during cultivation were diluted with water, centrifuged at 20,238 × g for 15 min, and the resulting supernatant was analyzed by reversed-phase HPLC.

For quantification of l-tyrosine and tyramine, 5 µL sample containing 100 µM cadaverine as internal standard was subjected to pre-column derivatization with *o*-phthaldialdehyde (OPA), and separated using a 40 × 4 mm LiChrospher 100 RP18 (5 μm) pre-column and a 125 × 4 mm LiChrospher 100 RP18 (5 μm) main column (CS-Chromatographie Service GmbH, Langerwehe, Germany) at 40 °C. As mobile phase, (A) 0.25% (v v^− 1^) sodium acetate (pH 6.0) and (B) methanol were used with a gradient and flow rates previously described [50]. The fluorescent derivatives were detected using a fluorescence detector (FLD G1321A, Agilent Technologies) with excitation and emission wavelengths of 230 and 450 nm, respectively.

To quantify *N*-acetyltyramine, *N*-propionyltyramine, which was quantified with an *N*-acetyltyramine standard, and tyrosol, 3 µL sample was injected on a system comprising a 40 × 4 mm LiChrospher 100 RP18 (5 μm) pre-column and a 125 × 4 mm CS-ODS 100 RP18 (5 μm) main column (CS-Chromatographie Service GmbH, Langerwehe, Germany) with a column temperature of 30 °C. As mobile phase, a gradient with (A) 0.1% (v v^− 1^) formic acid and (B) methanol as described previously [37] was employed at a constant flow rate of 0.8 mL min^− 1^. Detection was performed with a diode array detector (DAD G1321A, Agilent Technologies) at a wavelength of 224 nm for kinetic enzyme assay samples or at 280 nm for culture supernatants.

To quantify carbohydrates, 5 µL sample was injected on a 300 × 8 mm organic acid resin (10 μm) main column, coupled to a matching 40 × 8 mm pre-column (CS-Chromatographie Service GmbH, Langerwehe, Germany). Separation was performed under isocratic conditions with 5 mM sulfuric acid as mobile phase at a constant flow rate of 0.8 mL min^− 1^ and a column temperature of 60 °C for 17 min. Detection was performed using a refractive index detector (RID G1362A, Agilent Technologies).

The identities of *N*-acetyltyramine and *N*-propionyltyramine were confirmed by HPLC/MS analysis. Identity verification was performed using a combined SIM/SCAN method. In SIM mode, the quasi-molecular ion [M + H]^+^ of *N*-acetyltyramine was monitored at m/z 180. In addition, SCAN mode (m/z 110–280) was used to further confirm the identity of *N*-propionyltyramine. For sample preparation, culture supernatants were diluted 1:2 with acetonitrile and incubated at room temperature for 30 min to allow protein precipitation. Samples were then centrifuged at 20,238 × g for 15 min, and the resulting supernatant was collected for analysis. Mass spectrometric analysis was performed using an electrospray ionization (ESI) source coupled to an Agilent Infinity II 1290 system. The system was equipped with a flexible pump (G7104A, Agilent Technologies), a multisampler with dual needle (G7167B, Agilent Technologies), a diode array detector (DAD, G7117B, Agilent Technologies) fitted with a 10 mm Max-Light Cartridge Cell (G4212-60008, Agilent Technologies), and an InfinityLab LC/MSD iQ mass selective detector (G6160A, Agilent Technologies). A 1 µL sample was injected and separated on a 2.1 × 50 mm Infinity Poroshell 120 EC-C18 column (2.7 μm), coupled to a matching 2.1 × 5 mm pre-column (2.7 μm). Chromatographic separation was carried out at 40 °C with a constant flow rate of 0.8 mL min^− 1^. The mobile phase consisted of (A) 0.1% (v v^− 1^) formic acid in water and (B) acetonitrile. The following gradient was applied: starting at 95% A, decreasing to 40% A over 5 min, then to 10% A over 0.15 min, followed by a hold at 10% A for 0.6 min. The system was then returned to 95% A over 0.5 min, and equilibrated for 0.75 min.

## Results

### Physiological response of *C. glutamicum* to *N*-acetyltyramine

**Fig. 2:**
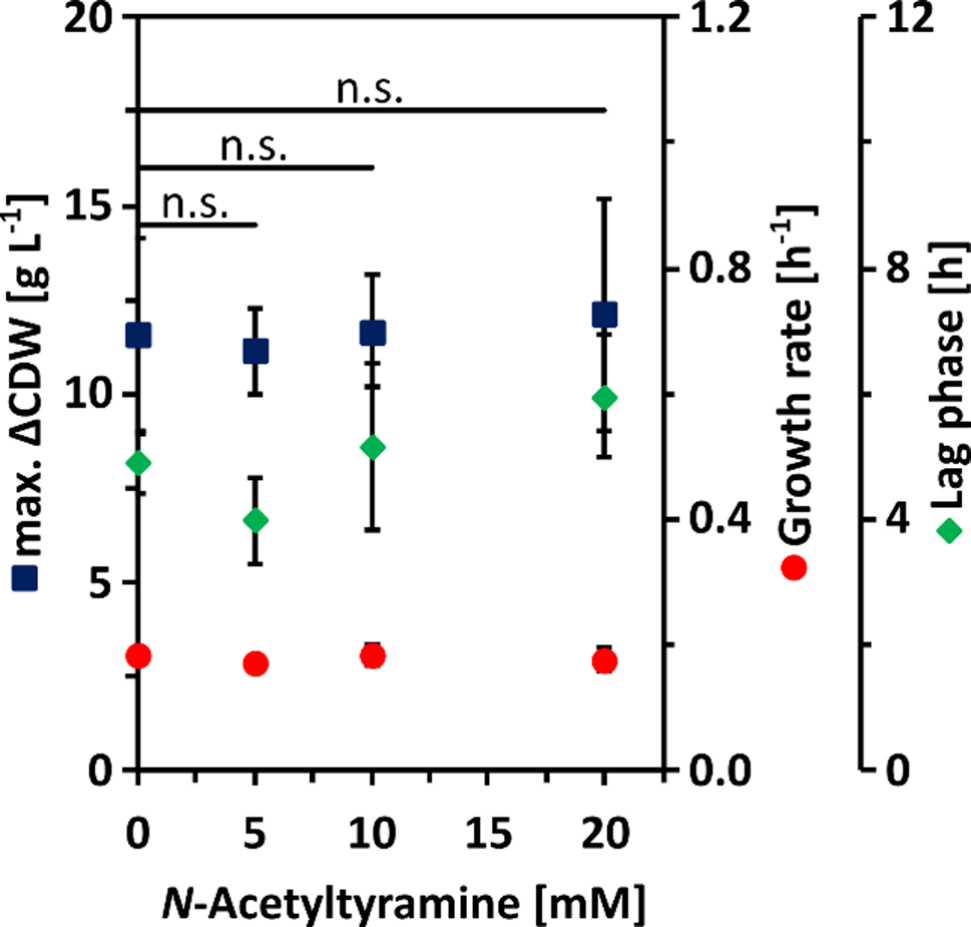
Growth parameters of *C. glutamicum* AROM3 in the presence of different supplemented *N*-acetyltyramine concentrations. Strain AROM3 was cultivated for 48 h in shake flasks in 10 mL CGXII minimal medium containing 40 g L^-1^ glucose, 0.5 mM l-phenylalanine, and *N*-acetyltyramine at concentrations ranging from 0 to 20 mM in shake flasks. Max. ∆cell dry weight (CDW, blue squares), growth rate (red circles), and lag phase (green diamonds) were determined for each *N*-acetyltyramine concentration. Values and error bars represent means and standard deviations from triplicate cultivations. No significant differences (n.s.) were observed relative to the control without *N*-acetyltyramine (0 mM) according to a two-sided Student’s *t*-test for all three growth parameters (*p* > 0.05)

*N*-Acetyltyramine has been shown to be active against diverse multidrug-resistant bacteria [3] with its antibacterial effect being thought to rely on the inhibition of quorum sensing [4]. Although quorum-sensing mechanisms have not been described for *C. glutamicum* and its robustness against a variety of aromatic compounds has been demonstrated [18], we tested potential toxic or inhibitory effects of *N*-acetyltyramine on the growth of *C. glutamicum*. The *C. glutamicum* strain AROM3 was chosen as base strain, since it has been engineered for the overproduction of the amino acid l-tyrosine that is an *N*-acetyltyramine precursor [24]. Strain AROM3 was cultivated for 48 h in glucose minimal medium containing 0–20 mM (0–3.6 g L^− 1^) *N*-acetyltyramine. No significant effect on the growth of strain AROM3 was observed at the tested *N*-acetyltyramine concentrations (Fig. 2), which were up to 300-times higher than the reported MIC of *N*-acetyltyramine for *S. aureus* or *P. aeruginosa* [3], underscoring the high robustness of *C. glutamicum*. However, aiming for an industrial-scale production of *N*-acetyltyramine, further investigations of the effects of higher *N*-acetyltyramine concentrations, as well as strain engineering to enable robust production at elevated titers, will be required.

In addition, degradation of *N*-acetyltyramine by strain AROM3 was tested. *N*-Acetyltyramine concentrations quantified by HPLC at the beginning and end of cultivation did not decrease (Supplementary Figure S1), indicating that *N*-acetyltyramine is stable under cultivation conditions and is neither degraded nor derivatized by the base strain AROM3. Taken together, strain AROM3 is a suitable host for *N*-acetyltyramine production at the gram-per-liter scale.

### Assaying bacterial and insect enzymes for acetylation of tyramine

*N-*Acetyltyramine can be synthesized from tyramine by acetylation of its amino group. However, *C. glutamicum* has not yet been described to encode an *N*-acetyltransferase that is active for aromatic substrates. To identify enzymes for the *N*-acetylation of tyramine, an in vitro assay was performed with candidate acetyltransferases. The bacterial arylamine *N*-acetyltransferase (NAT; EC 2.3.1.5) from *Mesorhizobium japonicum* MAFF 303,099 (NAT1_*Mj*_) is a suitable candidate for tyramine acetylation due to its broad substrate specificity, accepting various aromatic substrates for *N*-acetylation [51]. Furthermore, an acyltransferase from *Mycobacterium smegmatis* (AcT_*Ms*_), a close relative of *C. glutamicum*, has been shown to catalyze the trans-acetylation of a broad range of aliphatic and aromatic alcohols using ethyl acetate [52, 53]. To test the hypothesis that AcT_*Ms*_ may also *N*-acetylate tyramine using acetyl-CoA as acyl donor, it was included as a candidate in the in vitro assay. Insects use monoamine acetyltransferases for melatonin production, cuticle sclerotization, and inactivation of neurotransmitters [54]. An arylalkylamine *N*-acetyltransferase (AANAT; EC 2.3.1.87) from the silkworm *Bombyx mori* (AANAT_*Bm*_) has been demonstrated to be highly active with tyramine as a substrate [55]. A predicted dopamine *N*-acetyltransferase from *Acromyrmex echinatior* (AANAT_*Ae*_) shares 38% amino acid identity, and AlphaFold analysis combined with a structure alignment revealed a high predicted structural similarity in the arylalkylamine *N*-acetyltransferase domain with AANAT_*Bm*_ (Supplementary Figure S2). Thus, AANAT_*Bm*_ and AANAT_*Ae*_ were also chosen as candidate enzymes.

The activity of the four *N*-acetyltransferases was determined using crude extracts from *E. coli* DH5α strains carrying the plasmid for the overexpression of the respective acetyltransferase gene grown in LB medium. No *N*-acetyltyramine was detected for the crude extract from the empty vector control strain, demonstrating that endogenous acetyltransferases in the *E. coli* crude extract did not catalyze detectable acetylation of tyramine in the in vitro assay under the applied conditions (detection limit: 0.05 mM). In contrast, *N*-acetyltyramine synthesis from tyramine and acetyl-CoA was observed for all four enzyme producing strains (Supplementary Figure S3), indicating that these enzymes accepted acetyl-CoA and tyramine as substrates for *N*-acetylation. To the best of our knowledge, this is the first time *N*-acetylation activity is described for the predicted dopamine *N*-acetyltransferase AANAT_*Ae*_ as well as for AcT_*Ms*_, which has previously been characterized as an *O*-acetyltransferase. Crude extracts with AANAT_*Bm*_ exhibited the highest activity (5.7 ± 4.0 mU mg^− 1^ protein) among the tested enzymes (1.2 ± 0.6 mU mg^− 1^ protein for NAT1_*Mj*_, 1.9 ± 1.6 mU mg^− 1^ protein for AcT_*Ms*_, and 2.2 ± 0.8 mU mg^− 1^ protein for AANAT_*Ae*_). Within 3 h, an *N-*acetyltyramine concentration of 0.29 ± 0.03 mM was obtained with the crude extract containing AANAT_*Bm*_, indicating that approximately 58% of the acetyl-CoA in the in vitro assay was used for *N*-acetylation of tyramine. *N-*Acetylation of tyramine has previously been described for AANAT_*Bm*_ [55]. As expected, AANAT_*Bm*_ purified from a baculovirus overexpression system showed higher activity (about 70-fold) than the crude extract from the AANAT_*Bm*_-overexpressing *E. coli* strain tested here.

**Fig. 3:**
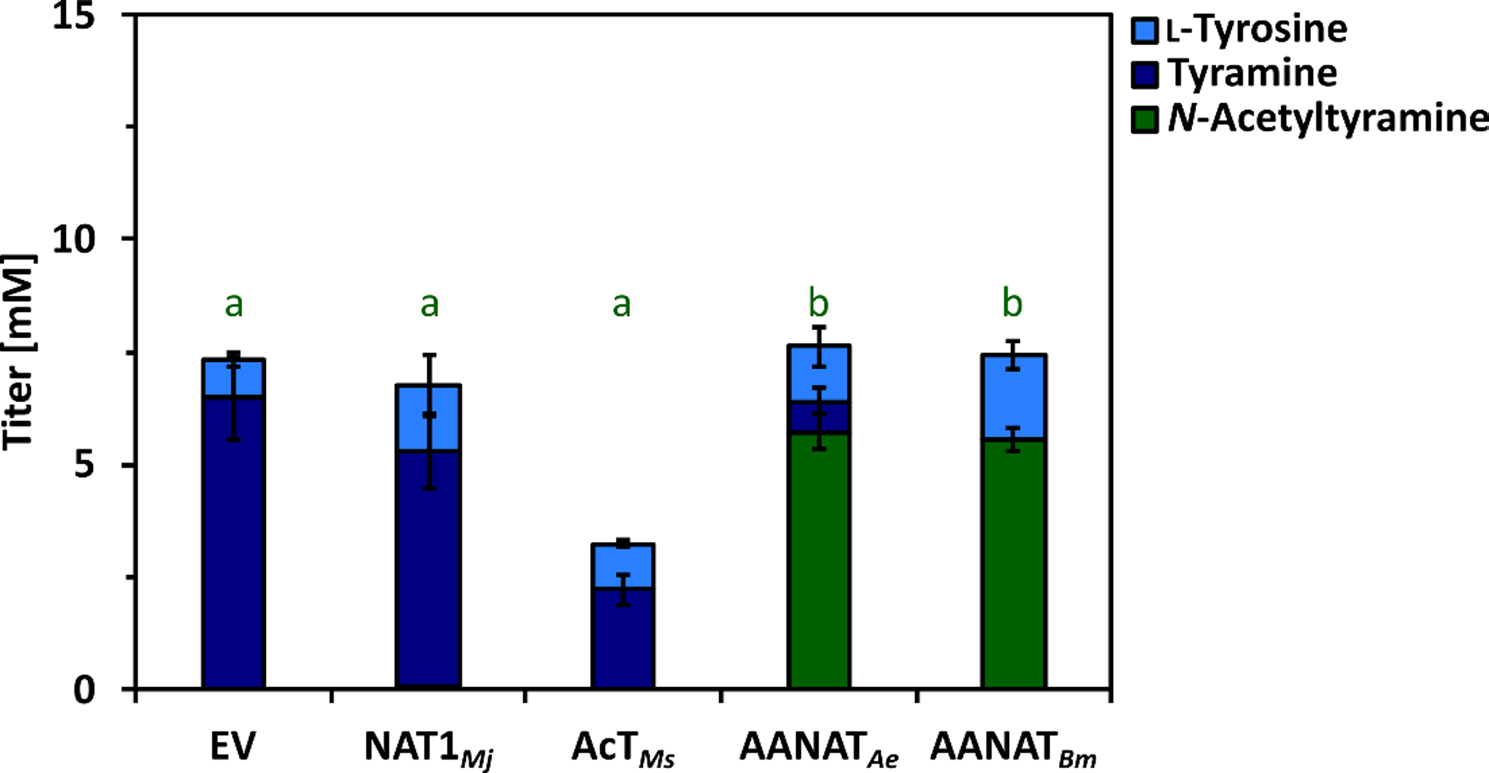
Comparison of different acetyltransferase genes for *de novo N*-acetyltyramine production. Titers of l-tyrosine (light blue), tyramine (dark blue), and *N*-acetyltyramine (green) by TRN strains carrying a pVWEx4-plasmid for the overproduction of the respective acetyltransferase indicated on the x-axis. The strains were cultivated for 120 h in the BioLector cultivation system in 1 mL CGXII minimal medium containing 40 g L^-1^ glucose and 0.5 mM l-phenylalanine. Glucose was depleted at the end of cultivation for all cultivations except for strains TRN (pVWEx4-AcT_*Ms*_) and TRN (pVWEx4-AANAT_*Bm*_). Values and error bars represent means and standard deviations from triplicate cultivations. Significance was calculated for *N*-acetyltyramine titers with an ANOVA followed by a Tukey’s honestly significant difference (HSD) test with α = 0.05; *p* = 6.5e^-12^

Since *N-*acetyltyramine formation was observed for all four candidate enzymes, the corresponding genes were evaluated for *de novo* production of *N-*acetyltyramine by *C. glutamicum*. The tyramine overproducing strain TRN [23] was therefore transformed with a plasmid for the overexpression of each acetyltransferase gene or an empty vector control. Upon cultivation for 120 h in a BioLector microcultivation system in CGXII minimal medium containing 40 g L^− 1^ glucose and 0.5 mM l-phenylalanine, *N*-acetyltyramine was not detected for the empty vector carrying control (Fig. 3) indicating that, under the chosen conditions, *C. glutamicum* strain AROM3 lacks acetyltransferase activity accepting tyramine as a substrate. Additionally, no *N*-acetyltyramine was detected in the culture supernatants of the AcT_*Ms*_ overproducing strain, and tyramine production by this strain was significantly reduced. In contrast, an additional peak was detected in the HPLC chromatograms of the culture supernatants of the other three strains, and its retention time and mass spectrum matched that of an *N*-acetyltyramine standard (Supplementary Figure S4). The *N*-acetyltyramine titer obtained for strain TRN (pVWEx4-NAT1_*Mj*_) was below 0.1 mM, whereas significantly higher titers were achieved upon the overexpression of the genes encoding the insect acetyltransferase AANAT_*Ae*_ or AANAT_*Bm*_ (5.7 ± 0.4 mM and 5.5 ± 0.3 mM, respectively). While 0.7 ± 0.3 mM tyramine remained unconverted in the supernatant of strain TRN (pVWEx4-AANAT_*Ae*_), tyramine was completely acetylated by strain TRN (pVWEx4-AANAT_*Bm*_), identifying AANAT_*Bm*_ as the most promising acetyltransferase for optimizing *de novo* production of *N*-acetyltyramine.

### Adjusting the plasmid-based expression strength of AANAT_*Bm*_

l-Tyrosine accumulated in the supernatant of *C. glutamicum* strain TRN (pVWEx4-AANAT_*Bm*_) to 1.9 ± 0.3 mM at the end of cultivation, indicating that l-tyrosine decarboxylation is a bottleneck in *N*-acetyltyramine production (Fig. 3). As demonstrated in previous studies, while strain TRN almost completely decarboxylated l-tyrosine to tyramine, addition of a second plasmid, independent of the insert, resulted in increased l-tyrosine accumulation [23, 37]. We assumed that these observations relied on an increased metabolic burden caused by the second plasmid reducing *tdc*_*Lb*_ expression, and that lowering the copy number of the second plasmid would improve *tdc*_*Lb*_ expression, resulting in a more complete conversion of l-tyrosine to tyramine and therefore higher *N*-acetyltyramine titers. To test this hypothesis, *N*-acetyltyramine production by TRN strains transformed with the plasmids pVWEx4, pVWEx1, or pSJEx3 for expression of the gene encoding AANAT_*Bm*_ was assessed. These plasmids share the promoter P_*tac*_ but differ in their origin of replication and, consequently, in their plasmid copy number and gene dosage [41]. The origin of replication pBL1, present in pSJEx3, has the lowest copy number of these three plasmids, followed by pHM1519 in pVWEx1. pVWEx4 contains a high-copy number variant of pHM1519 and thus has the highest copy number of these three plasmids [36, 39, 41].

**Fig. 4:**
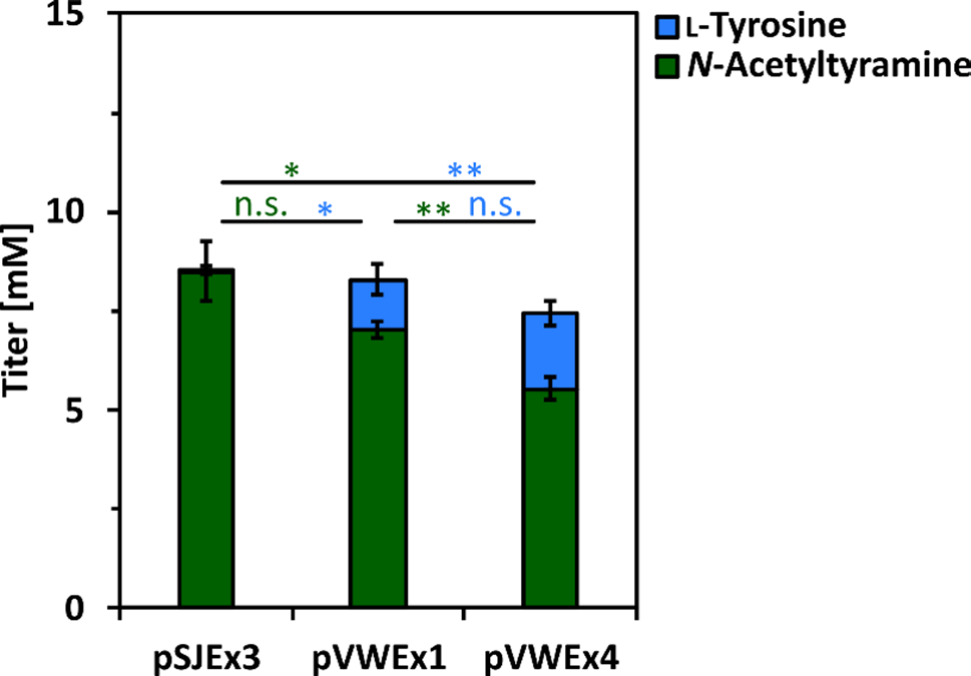
Comparison of plasmids supporting different gene dosages for the AANAT_*Bm*_ gene for *N*-acetyltyramine production. Titers of l-tyrosine (light blue) and *N*-acetyltyramine (green) by TRN strains carrying either pSJEx3-, pVWEx1-, or pVWEx4-AANAT_*Bm*_ as indicated on the x-axis. The gene dosage of the AANAT_*Bm*_-vectors increases from left to right. The strains were cultivated for 120 h in the BioLector cultivation system in 1 mL CGXII minimal medium containing 40 g L^-1^ glucose and 0.5 mM l-phenylalanine. Glucose was depleted at the end of cultivation for all cultivations. Values and error bars represent means and standard deviations from triplicate cultivations. Significance was calculated using a two-sided Student’s *t*-test with n.s.: *p* > 0.05, *: *p* < 0.05, **: *p* < 0.01

Reducing the plasmid copy number and the gene dosage of the AANAT_*Bm*_ gene increased *N*-acetyltyramine production and decreased l-tyrosine accumulation (Fig. 4), with a 27% higher *N*-acetyltyramine titer upon exchanging the high-copy number plasmid pVWEx4-AANAT_*Bm*_ with pVWEx1-AANAT_*Bm*_. These results align with our hypothesis that the l-tyrosine accumulation observed for TRN (pVWEx4-AANAT_*Bm*_) resulted from a reduced *tdc*_*Lb*_ expression due to the metabolic burden caused by the second plasmid, and that this can be overcome by choosing a plasmid with a lower copy number for expression of the gene encoding AANAT_*Bm*_. Since the *N*-acetyltyramine titer achieved with pSJEx3-AANAT_*Bm*_ did not increase significantly compared to pVWEx1-AANAT_*Bm*_, and to avoid that AANAT_*Bm*_ gene expression becomes limiting, we proceeded with strain TRN (pVWEx1-AANAT_*Bm*_) – hereinafter referred to as *C. glutamicum* strain ATRN – for subsequent media optimization.

### Design of experiments-guided media optimization

Optimization of the media composition has recently proven an effective strategy for improving the biosynthesis of shikimate-pathway-derived products [26, 34]. Accordingly, a DoE approach was applied to identify media components that significantly influence *N*-acetyltyramine production by *C. glutamicum* strain ATRN, followed by optimizing their concentrations to improve *N*-acetyltyramine production.

#### Screening design to identify media components significantly affecting *N*-acetyltyramine production

To identify media components with a significant effect on *N*-acetyltyramine production, a central composite design (CCD) was used. Because testing multiple concentrations of the 14 CGXII minimal medium components [36] plus the l-phenylalanine required by the bradytrophic strain ATRN in a full factorial design was not feasible, a fractional factorial design was used for the initial screening. The number of experimental runs was substantially reduced by confounding 10 components with the combined effects of the remaining five (Supplementary Table S3), reducing the number of cube portions to be tested by 99.9% (from 32768 to 32).

The resulting screening model identified the following eight candidate media components for further testing (*p* < 0.05): glucose, l-phenylalanine, MOPS, MnSO_4_, phosphate, CaCl_2_, biotin, and urea (Table 3). To predict the optimal concentration of these candidate media components and to test for two-factor interactions, response surface methodology (RSM) was employed in the next step. To this end, we aimed to test up to six components in a fractional factorial design to balance the need for model accuracy with experimental feasibility.

**Table 3:** ANOVA analysis results of the screening design predicting the effects on the *N*-acetyltyramine titer

Factor	t-value	Prob > t
Intercept	6.49	< 0.001
Block	-2.42	0.024
Ammonium sulfate	-0.70	0.492
Urea	-4.54	< 0.001
Phosphate	0.97	0.341
MOPS	3.30	0.003
Glucose	7.72	< 0.001
CaCl_2_	-2.10	0.048
MgSO_4_	0.86	0.399
FeSO_4_	1.80	0.085
MnSO_4_	2.44	0.023
ZnSO_4_	1.29	0.211
CuSO_4_	-0.31	0.757
NiCl_2_	0.38	0.704
PCA	1.47	0.156
Biotin	-2.47	0.022
l-Phenylalanine	4.29	< 0.001
Ammonium sulfate^2^	0.33	0.745
Urea^2^	-1.04	0.309
Phosphate^2^	-2.53	0.019
MOPS^2^	-1.59	0.126
Glucose^2^	2.38	0.026
CaCl_2_^2^	-1.38	0.180
MgSO_4_^2^	-0.55	0.589
FeSO_4_^2^	-0.43	0.674
MnSO_4_^2^	-0.44	0.667
ZnSO_4_^2^	-0.87	0.396
CuSO_4_^2^	0.03	0.974
NiCl_2_^2^	-0.14	0.889
PCA^2^	-1.62	0.119
Biotin^2^	-0.20	0.841
l-Phenylalanine^2^	1.34	0.194
	**F-value**	**Prob > F**
First order	9.02	< 0.001
Quadratic effects	2.31	0.036
Lack of fit	18.96	0.002

The effect of each factor and its probabilities, as well as the F-values and probabilities of the model are given. Squared factors indicate quadratic effects. Two-factor interactions were excluded since the strong confounding among the media components did not allow for reliable predictions

As expected, the concentration of glucose as the limiting carbon source positively correlated with the *N*-acetyltyramine titer. However, the *N*-acetyltyramine substrate yield Y_P/S_ was not significantly affected by glucose (Supplementary Table S6). Consequently, glucose was excluded from further media refinement experiments. The effect of urea increasing the pH value of cultures due to release of ammonia during its utilization has been previously described [56]. This effect might be enhanced at low concentrations of MOPS and the phosphate buffer, which is reflected by an increase in pH to 8–9 observed in most cultivations that combined increased urea concentrations with decreased concentrations of MOPS and phosphate (data not shown), indicating that the predicted effects of MOPS and phosphate are likely pH-driven. When transferring the *N*-acetyltyramine production into industrial scale in the future, the pH control during fermentation processes does not necessitate an optimization of these buffers. Therefore, both components were omitted from the next design.

After excluding glucose, MOPS, and phosphate as components for further optimization, we were able to include one more medium component for testing in the CCD. Although FeSO_4_ was not identified as a significant factor by the screening model (*p* = 0.085), it is involved in a variety of energy metabolism processes of *C. glutamicum*. For instance, it is an essential component of respiratory chain enzymes [57, 58], and affects TCA cycle gene expression [59, 60]. Moreover, iron contributes to the regulation of thiamine pyrophosphate (TPP) biosynthesis and is therefore assumed to affect the oxidative decarboxylation of pyruvate to acetyl-CoA catalyzed by the pyruvate dehydrogenase (Pdh) [61]. Similar to *N*-acetyltyramine, carotenoid and terpene production depends on sufficient acetyl-CoA availability, and refinement of the FeSO_4_ concentration in the medium was demonstrated to benefit carotenoid and terpene production by *C. glutamicum* [14, 15]. Therefore, the FeSO_4_ concentration was included in the RSM and steepest ascent analysis.

#### RSM and steepest ascent analysis to predict optimal concentrations of candidate media components

After having identified urea, CaCl_2_, FeSO_4_, MnSO_4_, biotin, and l-phenylalanine as candidate media components for media optimization for *N*-acetyltyramine production, a fractional factorial design confounding l-phenylalanine with the combined effect of the other five components (Supplementary Table S4) was used to investigate their influence on *N*-acetyltyramine production in more detail. The resulting response surface model showed no significant two-factor interactions, but identified urea and l-phenylalanine as significant factors affecting *N*-acetyltyramine production, with urea exhibiting the most dominant effect (Supplementary Table S7).

**Fig. 5:**
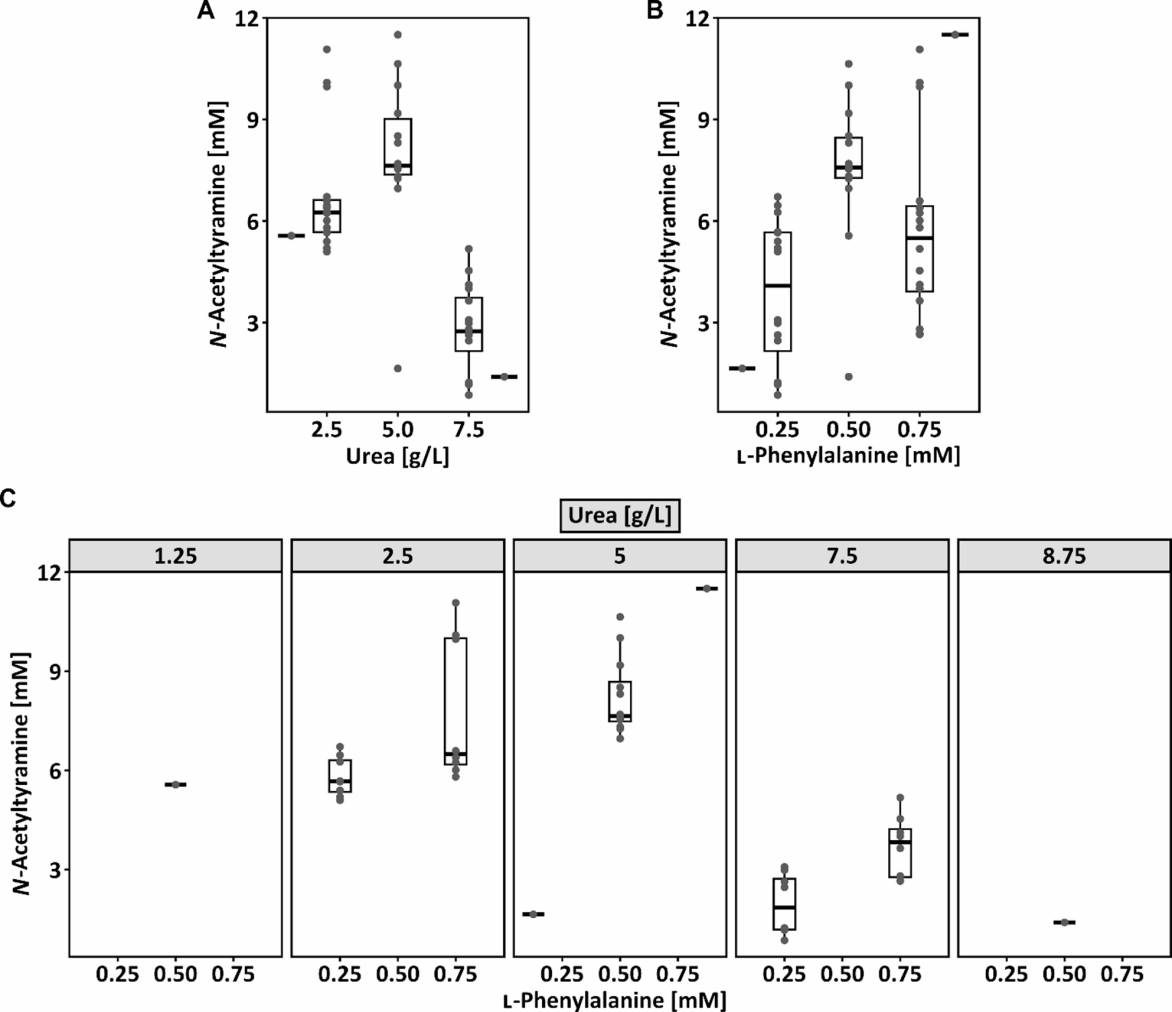
Boxplots of media components significantly affecting *N*-acetyltyramine production identified by RSM. *N*-Acetyltyramine titers produced by strain ATRN are illustrated as a dependence on the urea (**A**) or l-phenylalanine (**B**) concentrations tested in the RSM. To visualize the linear positive effect of l-phenylalanine, results were clustered by the urea concentration of the respective cultivations (**C**). Grey circles represent the values measured for each single cultivation

For urea, both first-order and quadratic effects were significant, as reflected by a decline in *N*-acetyltyramine titers at urea concentrations deviating from the center point, particularly at higher urea concentrations (Fig. 5A). Although the response surface model predicted a first-order effect for l-phenylalanine, the corresponding boxplot suggested the presence of an additional quadratic component (Fig. 5B), likely due to the dominant influence of urea masking the effect of l-phenylalanine. To disentangle the two effects, the *N*-acetyltyramine titers obtained at different l-phenylalanine concentrations were grouped according to the urea concentration of the respective cultures, clearly revealing a positive linear correlation for l-phenylalanine (Fig. 5C).

The stationary point of the response surface model predicted negative, thus impossible, concentrations of CaCl_2_ and FeSO_4_. Consequently, a steepest ascent analysis was conducted to identify the urea, CaCl_2_, FeSO_4_, MnSO_4_, biotin, and l-phenylalanine concentrations predicted to yield the strongest increase in *N*-acetyltyramine titer according to the response surface model. The first five media compositions at distances between 0.5 and 2.5 from the center point composition along the path of steepest ascent (Supplementary Table S5) were evaluated in a BioLector cultivation, along with the center point corresponding to the standard CGXII minimal medium composition.

**Fig. 6:**
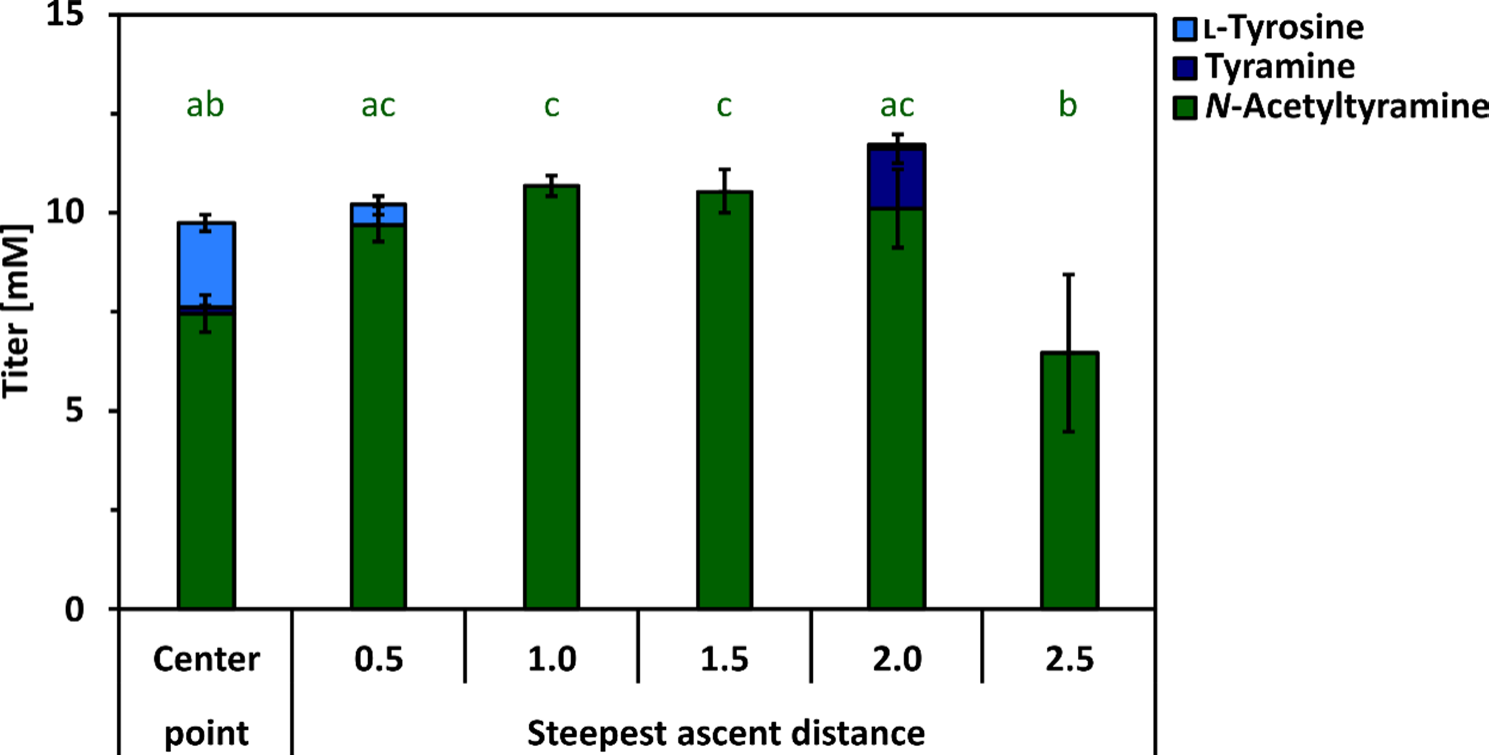
*N*-Acetyltyramine production at media compositions according to the path of steepest ascent analysis. Titers of l-tyrosine (light blue), tyramine (dark blue), and *N*-acetyltyramine (green) by strain ATRN are indicated. Strain ATRN was cultivated for 120 h in the BioLector cultivation system in 1 mL CGXII minimal medium containing 40 g L^-1^ glucose. The medium composition at the center point corresponds to the standard composition of the CGXII minimal medium. Glucose was depleted at the end of cultivation for all cultivations. Values and error bars represent means and standard deviations from triplicate cultivations. Significance was calculated for *N*-acetyltyramine titers with an ANOVA followed by a Tukey’s honestly significant difference (HSD) test with α = 0.05; *p* = 0.00059

*N*-Acetyltyramine titers increased along the path of steepest ascent and reached a maximum of 10.7 ± 0.3 mM at a distance of 1.0 (Fig. 6). Titers remained similar for the next two tested distances but declined markedly at a distance of 2.5. This probably resulted from the FeSO_4_ concentration being only 6% of the concentration in the standard CGXII minimal medium, impairing growth and consequently production. This was evident in the lower growth rate observed at this distance compared with the other tested medium compositions (Supplementary Figure S5). While neither l-tyrosine nor tyramine were detected (< 0.05 mM) at distances 1.0 and 1.5, reflecting their complete conversion to *N*-acetyltyramine, tyramine accumulated at a distance of 2.0 to considerable concentrations (1.5 ± 0.4 mM). Iron shortage is presumed to reduce thiamine pyrophosphate (TPP) synthesis, thereby impairing the TPP-dependent pyruvate dehydrogenase Pdh that catalyzes the oxidative decarboxylation of pyruvate to acetyl-CoA (Fig. 9) [61]. Consequently, the 72% lower iron concentration at the steepest ascent distance 2.0 may have limited acetyl-CoA availability, rendering tyramine acetylation rate-limiting and thereby causing its accumulation under this condition. Overall, at a distance of 1.0 along the path of steepest ascent, the *N*-acetyltyramine production increased by 43% compared to the center point. Furthermore, the lowest standard deviation in *N*-acetyltyramine titers was obtained for this refined medium composition at distance 1.0 (Supplementary Table S5), indicating its robustness and benefit for *N*-acetyltyramine production.

**Fig. 7:**
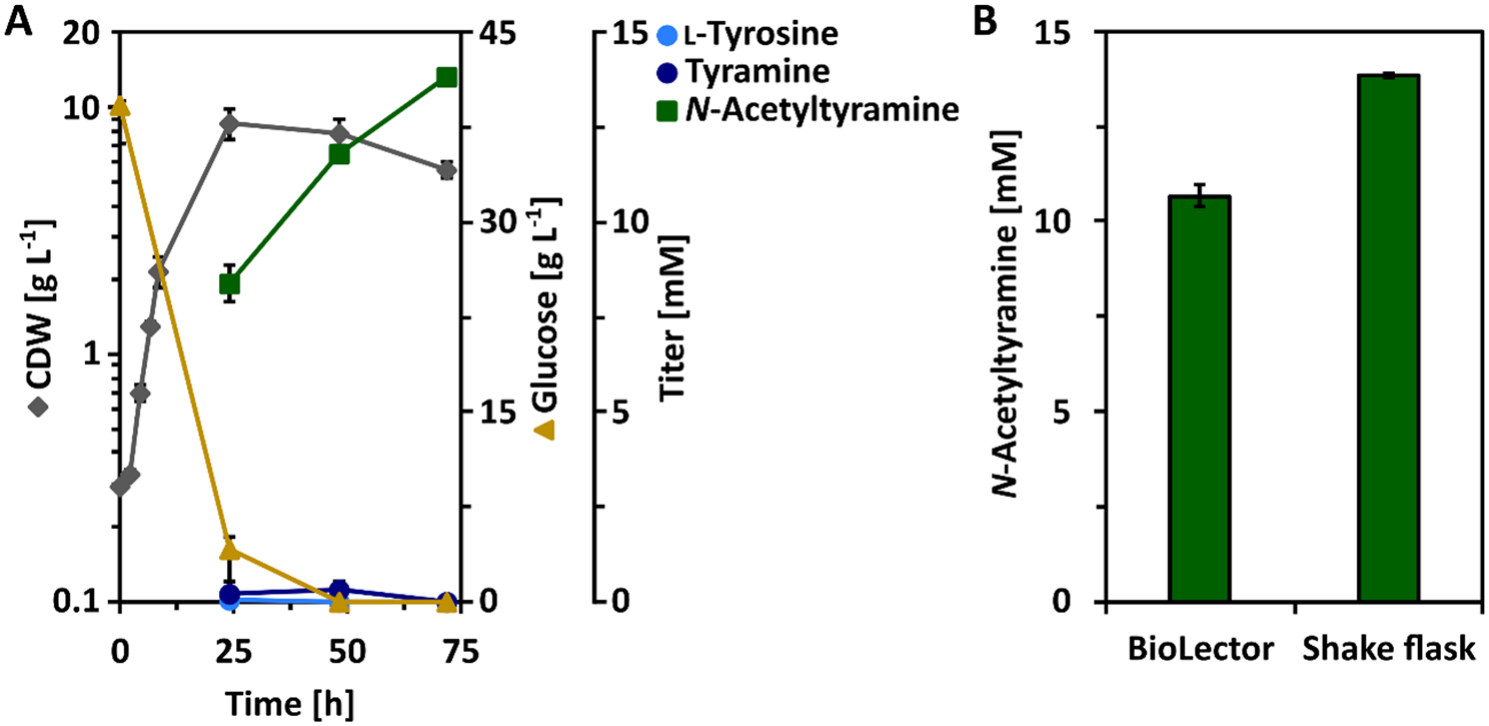
*N*-Acetyltyramine production during shake flask cultivation by strain ATRN in optimized CGXII medium. CDW (grey diamonds), glucose (yellow triangles), and extracellular l-tyrosine (light blue circles), tyramine (dark blue circles), and *N*-acetyltyramine (green squares) concentrations measured over time during shake flask cultivation (A) are depicted. Strain ATRN was cultivated for 72 h in shake flasks in 10 mL optimized CGXII minimal medium containing 40 g L^-1^ glucose. *N*-Acetyltyramine titers obtained after cultivation in optimized CGXII minimal medium for 120 h in the BioLector cultivation system or after 72 h in shake flasks are compared in B. No l-tyrosine or tyramine were detected at the end of these cultivations. Values and error bars represent means and standard deviations from triplicate cultivations

To assess the time course of *N*-acetyltyramine production by strain ATRN in the refined medium, the production process was transferred to shake flask cultivation with a culture volume of 10 mL, and samples were collected at regular intervals for HPLC analysis. Extracellular l-tyrosine and tyramine concentrations remained below 0.1 mM and 0.3 mM during the cultivation, respectively, and were completely depleted by the end of cultivation, indicating their efficient conversion (Fig. 7A). *N*-Acetyltyramine accumulated in the culture supernatant throughout the cultivation, reaching a final titer of 13.8 ± 0.1 mM (2.5 ± 0.1 g L^− 1^), reflecting an almost 30% increased compared to the production in the BioLector (Fig. 7B). Furthermore, the cultivation time was reduced to 72 h, resulting in a volumetric productivity of 34.4 ± 0.1 mg L^− 1^ h^− 1^. These results demonstrate the stable transfer of *N*-acetyltyramine production to shake flask cultivation.

#### Transfer of the optimized medium to the production of other l-tyrosine derivatives

**Fig. 8:**
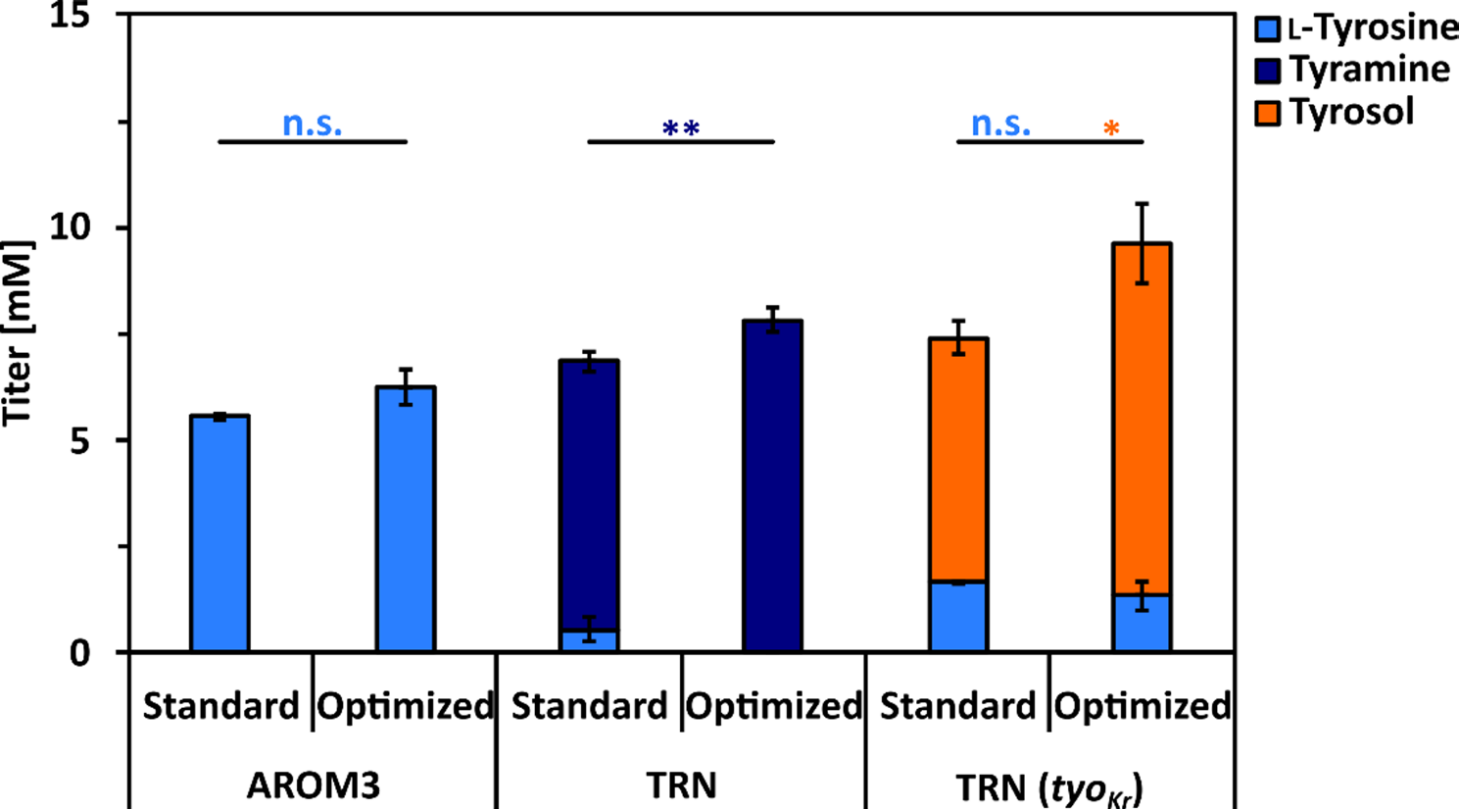
Production of l-tyrosine and derivatives in optimized CGXII minimal medium. Titers of l-tyrosine (light blue), tyramine (dark blue), and tyrosol (orange) by the strains AROM3, TRN, and TRN (*tyo*_*Kr*_) are indicated. The strains were cultivated for 120 h in the BioLector cultivation system in 1 mL CGXII minimal medium in the standard or optimized composition containing 40 g L^-1^ glucose. Glucose was depleted at the end of cultivation for all cultivations. Values and error bars represent means and standard deviations from triplicate cultivations. Significance was calculated using a two-sided Student’s *t*-test with n.s.: *p* > 0.05, *: *p* < 0.05, **: *p* < 0.01

To test whether the optimized medium benefits the production of l-tyrosine and its derivatives beyond *N*-acetyltyramine, the overproduction of l-tyrosine, tyramine, and tyrosol in the optimized CGXII minimal medium by the previously established strains AROM3 [24], TRN [23], and TRN (*tyo*_*Kr*_) [37] was examined. The l-tyrosine titer did not differ significantly between the standard and optimized CGXII minimal medium (Fig. 8). In contrast, the optimized medium increased the tyramine titer by 24% (from 6.3 ± 0.2 mM to 7.8 ± 0.3 mM) and the tyrosol titer by 44% (from 5.8 ± 0.4 mM to 8.3 ± 0.9 mM). Thus, the CGXII minimal medium optimized for *N*-acetyltyramine production was successfully applied to enhance the production of further l-tyrosine derivatives.

### Expanding the product spectrum to *N*-propionyltyramine

**Fig. 9:**
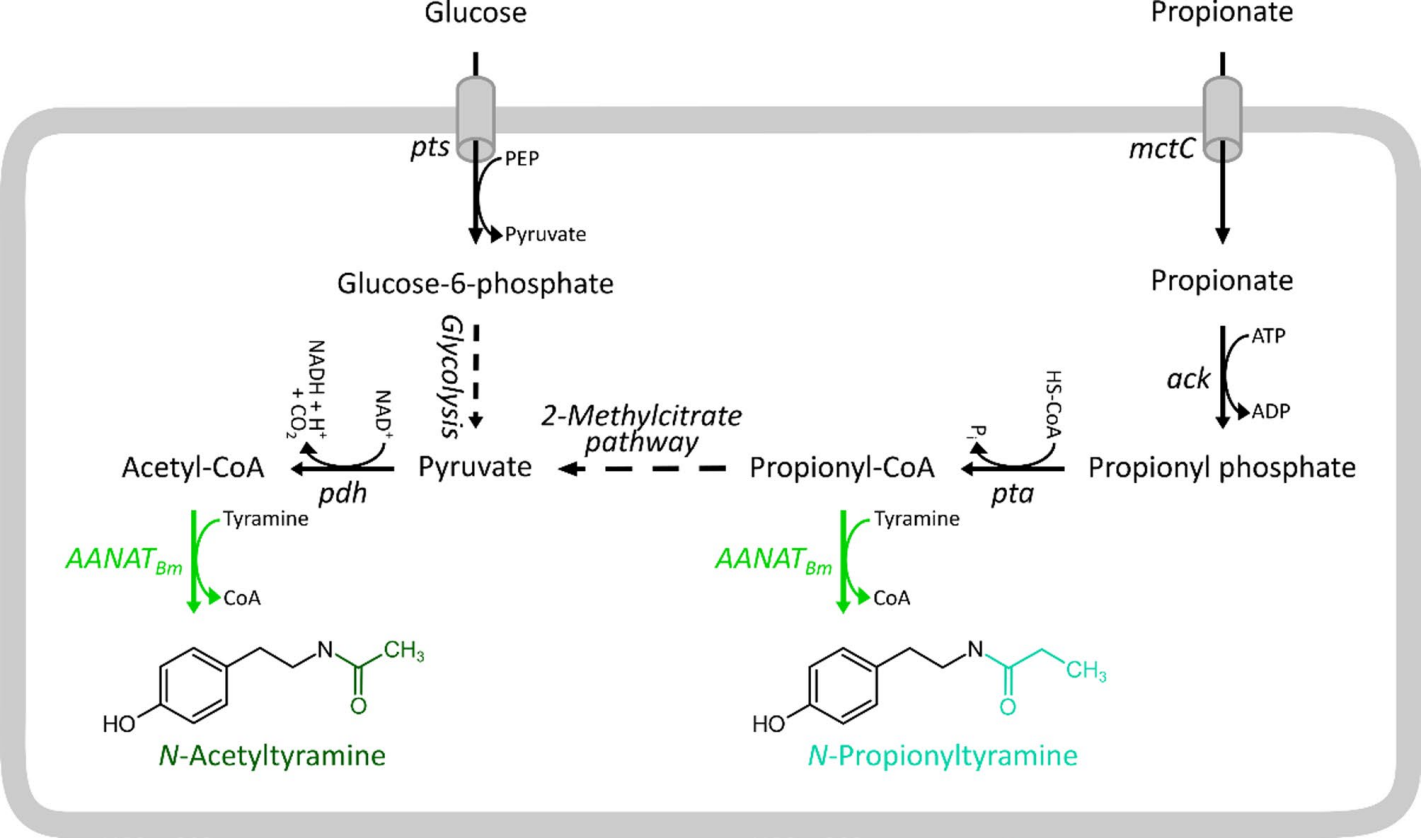
Biosynthesis pathway of *N*-acetyltyramine and *N*-propionyltyramine from glucose and propionate as carbon sources. Dashed arrows indicate multiple reaction steps. Plasmid-based overexpression of the gene encoding AANAT_*Bm*_ is indicated in green. Genes encoding transporters or enzymes catalyzing the corresponding reactions include: *AANAT*_*Bm*_: arylalkylamine *N*-acetyltransferase from *Bombyx mori*, *ack*: acetate kinase, *mctC*: monocarboxylic acid uptake system, *pta*: phosphotransacetylase, *pdh*: pyruvate dehydrogenase, *pts*: phosphotransferase uptake system. Abbreviations: ATP: adenosine triphosphate, ADP: adenosine diphosphate, CoA: coenzyme A, NAD: nicotinamide adenine dinucleotide, PEP: phosphoenolpyruvate, P_i_: inorganic phosphate

In the established *N*-acetyltyramine production, the acetyl-CoA required for tyramine acetylation is derived from the oxidative decarboxylation of pyruvate formed during glycolysis (Fig. 9). To expand the product spectrum, an alternative acyl donor for tyramine functionalization that *C. glutamicum* can natively synthesize was tested. Propionyl-CoA is an intermediate of propionate catabolism in *C. glutamicum*. Propionate is imported via the monocarboxylic acid uptake system MctC [62], followed by its phosphorylation via the phosphotransacetylase Pta, and activation to propionyl-CoA by the acetate kinase Ack [63]. Propionyl-CoA is subsequently converted into pyruvate via the 2-methylcitrate pathway [64]. Therefore, it was assessed whether the propionyl-CoA formed during propionate utilization can serve as an acyl donor to drive *N*-propionylation of tyramine by AANAT_*Bm*_ in vivo.

#### Assaying AANAT_*Bm*_ for the *N*-acetylation and *N*-propionylation of tyramine

Some insect AANAT enzymes, such as the AANAT from *Tribolium castaneum*, have been demonstrated to accept propionyl-CoA for the acylation of aromatic amines [65], and another AANAT enzyme from *Bombyx mori* (AANAT3) has been shown to accept the short-chain CoA thioesters malonyl-CoA and butyryl-CoA as substrates [66]. However, the AANAT_*Bm*_ used in strain ATRN has not yet been evaluated regarding its activity with propionyl-CoA, although it has previously been shown to accept various long-chain CoA thioesters for the acylation of tryptamine [67]. Therefore, the activity of AANAT_*Bm*_ for the *N*-propionylation of tyramine was assessed.

To determine the apparent kinetic parameters of AANAT_*Bm*_ for tyramine acylation, enzyme activity was measured at varying acetyl-CoA and propionyl-CoA concentrations using an in vitro assay with crude extract from an *E. coli* DH5α strain overexpressing AANAT_*Bm*_. Activity was observed with both CoA thioesters as substrates (Supplementary Figure S6). The apparent kinetic data obtained for propionyl-CoA indicate substrate inhibition, with an apparent *K*_I_ value of 1.8 mM (Table 4). Although the maximal specific activity of AANAT_*Bm*_ determined for propionyl-CoA was 24% lower than for acetyl-CoA, its activity for *N*-propionylation of tyramine was successfully demonstrated in vitro, indicating its potential for in vivo* N*-propionyltyramine production.

**Table 4:** Apparent kinetic parameters of AANAT_*Bm*_ determined for acetyl-CoA and propionyl-CoA

Substrate	*K*_M, app_ [mM]	*K*_I, app_ [mM]	Max. specific activity [nmol min^− 1^ mg^− 1^ total protein]
Acetyl-CoA	0.08	-	77.1
Propionyl-CoA	0.14	1.83	101.0

Apparent kinetic parameters were determined from the initial rates measured at different acetyl-CoA and propionyl-CoA concentrations for *N*-acylation of tyramine by crude extract from *E. coli* DH5α carrying a plasmid for the overproduction of AANAT_*Bm*_. The maximum specific velocity was calculated as the apparent V_*max*_ divided by the total protein concentration used in the enzyme assay

#### *De novo* production of *N*-propionyltyramine

**Fig. 10:**
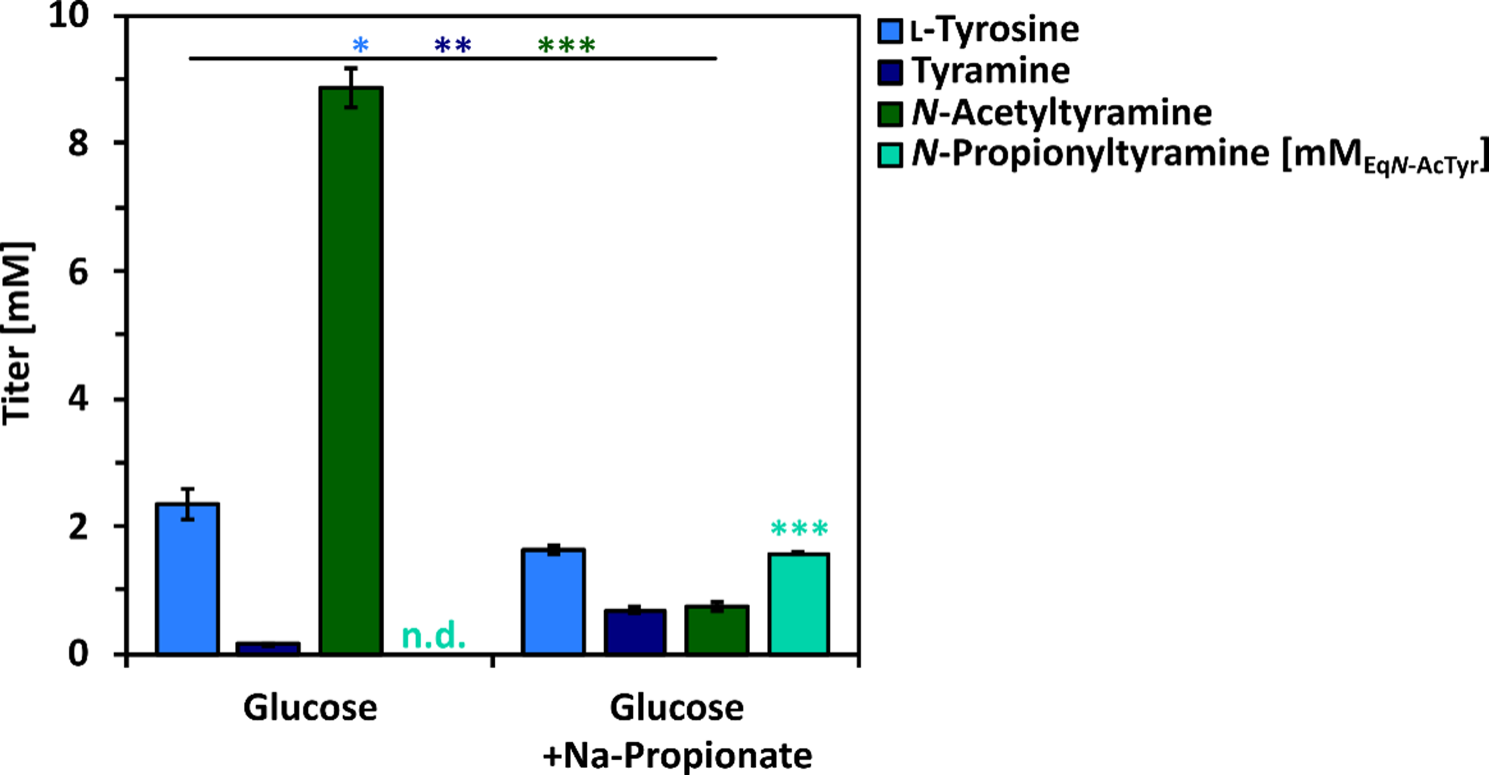
*De novo* production of *N*-acetyltyramine and *N*-propionyltyramine on the combined carbon sources glucose and propionate. Titers of l-tyrosine (light blue), tyramine (dark blue), *N*-acetyltyramine (dark green), and *N*-propionyltyramine (light green) by strain ATRN cultivated in the BioLector cultivation system in 1 mL CGXII minimal medium containing either 222 mM glucose (corresponding to 40 g L^-1^) or a combination of 111 mM glucose and 222 mM sodium propionate for 120 h. *N*-Propionyltyramine titers were quantified as equivalents with the *N*-acetyltyramine standard. Glucose was depleted at the end of cultivation for all cultures containing only glucose as carbon source, while 11.8 ± 2.4 mM glucose and 214 ± 5 mM Na-propionate remained in the supernatants of the cultures that contained a mixture of both carbon sources. Values and error bars represent means and standard deviations from triplicate cultivations. Significance was calculated for l-tyrosine, tyramine, and *N*-acetyltyramine using a two-sided Student’s *t*-test. For *N*-propionyltyramine production on the glucose and sodium propionate mixture, significance was calculated using a one-sample one-sided Student’s *t*-test against a reference value of 0. n.d.: not detected, *: *p *< 0.05, **: *p *< 0.01, ***: *p* < 0.001

To assess *de novo* production of *N*-propionyltyramine, strain ATRN was cultivated in CGXII minimal medium that contained either 222 mM glucose (corresponding to 40 g L^− 1^) as a control or a mixture of 111 mM glucose and 222 mM sodium propionate, corresponding to an equimolar carbon concentration of the co-substrates. *N*-Acetyltyramine production of 0.7 ± 0.1 mM on the glucose-propionate mixture was significantly lower compared to 8.9 ± 0.7 mM with glucose as the sole carbon source (Fig. 10), likely due to the inhibitory effects of propionate on *C. glutamicum* [64, 68]. However, an additional peak was detected in the HPLC chromatogram of the propionate-containing culture supernatant (Supplementary Figure S7A). The most intense signal in the mass spectrum of this peak, at m/z 194.1, matched the calculated value for protonated *N*-propionyltyramine [M + H]^+^ (Supplementary Figure S7B), indicating its successful production by strain ATRN in the presence of propionate. As no commercial *N*-propionyltyramine standard was available, *N*-propionyltyramine was quantified with an *N*-acetyltyramine standard, resulting in an estimated *N*-propionyltyramine titer of 1.6 ± 0.1 mM *N*-acetyltyramine equivalents (mM_Eq*N*-AcTyr_). Additionally, 1.6 ± 0.1 mM l-tyrosine and 0.7 ± 0.1 mM tyramine accumulated at the end of cultivation on the glucose-propionate mixture indicating the potential for further optimization to overcome their incomplete conversion to *N*-acetyltyramine or *N*-propionyltyramine under these conditions. Overall, these results demonstrate the potential for expanding the product spectrum of strain ATRN to other tyramine derivatives by supplementing alternative acyl-CoA substrates.

## Discussion

In this study, heterologous overexpression of *N*-acetyltransferase genes in a tyramine overproducing *C. glutamicum* strain enabled *de novo N*-acetyltyramine production. An in vitro assay evaluating bacterial and insect acetyltransferases demonstrated that all four tested enzymes catalyzed the *N*-acetylation of tyramine. However, in vivo, only 0.05 mM or no *N*-acetyltyramine was detected upon overexpression of the genes encoding the bacterial NAT1_*Mj*_ and AcT_*Ms*_, respectively (Fig. 3). These discrepancies might be attributed to the substrate specificities of these enzymes, resulting in a low activity toward tyramine in vivo, in contrast to the in vitro assay, in which tyramine was supplied as the sole substrate at a high concentration (25 mM). The bacterial acetyltransferase NAT1_*Mj*_ is thought to be involved in the detoxification of soil pollutants and may therefore has evolved a high specificity for substrates not including tyramine [51]. AcT_*Ms*_ has previously only been described for the *O*-acetylation of alcohols [53], and is therefore likely to prefer alcohols over tyramine as substrates in vivo. The *O*-acetylation of intracellular alcohols might have resulted in products that not only caused the lower growth rate observed for strain TRN (pVWEx4-AcT_*Ms*_) (Supplementary Figure S8), but might also have inhibited steps in the tyramine synthesis pathway, thereby reducing tyramine production by this strain.

The highest enzyme activities were observed for the insect AANAT enzymes, and overexpression of the corresponding genes resulted in significantly higher *N*-acetyltyramine titers compared to the bacterial acetyltransferase genes (Fig. 3). This may be attributed to the physiological roles of these enzymes. Insect AANAT enzymes are thought to be involved in the inactivation of amine neurotransmitters [54, 69], such as tyramine [70], and may thus have evolved high affinities towards these substrates, as reflected by the low *K*_M_ values in the submillimolar range described for AANAT_*Bm*_ for tryptamine [55] and as observed here for acetyl-CoA and propionyl-CoA (Table 4). The *N*-acetylation of tyramine by AANAT_*Ae*_ observed in this study confirms its previously only predicted annotation as an *N*-acetyltransferase. Given the high predicted structural similarity between the arylalkylamine *N*-acetyltransferase domains of AANAT_*Ae*_ and AANAT_*Bm*_ (Supplementary Figure S2), it is reasonable to assume that their substrate binding pockets and active sites are both similar, which may explain their shared activity toward tyramine. Further research is required to characterize AANAT_*Ae*_ in more detail, including assessing its acyl and aryl substrate spectrum as well as structural analysis, such as the identification of its substrate-binding site through enzyme activity assays using AANAT_*Ae*_ amino acid exchange mutants.

Assaying AANAT_*Bm*_ for both acetyl-CoA and propionyl-CoA has demonstrated its activity for propionyl-CoA as a short-chain acyl substrate, with a maximal specific activity reduced by 24% compared to acetyl-CoA (Table 2). This finding is consistent with previous studies that have reported a decrease in catalytic efficiency with increasing chain length of the CoA thioester substrate, which might be contributed to steric hindrance in the active site [65, 66, 71]. Acetylation by insect AANAT enzymes is described to be initiated by the binding of acetyl-CoA, which leads to conformational changes that open a hydrophobic cavity for binding of the aryl substrate. After acetyl transfer, binding of a new acetyl-CoA molecule releases the formed product [72]. The conformational changes that open the active site for the aryl substrate, as well as the interchange of the product with a new acyl-CoA molecule, might be impeded by an increased chain length of the acyl-CoA thioester. Substrate inhibition was shown here for propionyl-CoA, with an apparent *K*_I_ value of 1.8 mM. So far, inhibition of insect AANAT enzymes has only been described for long-chain CoA thioesters, which has been attributed to a hindrance in the correct orientation of the aryl substrate in the enzyme’s active site [65, 73]. Further structure-function studies are needed to better understand the activity of AANAT_*Bm*_ with different acyl-CoA substrates. These should include enzyme activity assays using the purified enzyme to determine its kinetic parameters for propionyl-CoA and to resolve the mechanism behind the substrate inhibition observed for AANAT_*Bm*_ at increased levels of propionyl-CoA and other CoA thioesters, which might be associated with an impeded release of the products [74].

Overexpression of the gene encoding AANAT_*Bm*_ in the tyramine overproducing strain TRN resulted in *de novo N*-acetyltyramine production. Balancing the expression strength of the *tdc*_*Lb*_ and AANAT_*Bm*_ gene combined with optimizing the culture medium was demonstrated to be a successful strategy for significantly enhancing production titers. Using a DoE approach for media optimization identified the urea concentration as the main factor, with its reduction significantly improving *N*-acetyltyramine production. Previous studies have reported that a decreased urea concentration enhances the production of the shikimate pathway-derived PCA by *C. glutamicum*, with the authors attributing this effect, among other factors, to pH perturbations at elevated urea concentrations [34]. *C. glutamicum* utilizes urea as a nitrogen source via an urease, which hydrolyzes urea and thereby releases ammonia, which potentially increases the medium pH value [56]. Although *C. glutamicum* possesses mechanisms for pH homeostasis, its internal pH value has been shown to rise under alkaline external conditions [59], reflecting the limitations of these mechanisms. Therefore, we hypothesize that the beneficial effect of reduced urea concentrations on *N*-acetyltyramine production observed in this study might be linked to its alterations in intracellular pH. This assumption is supported by lower pH values measured at the end of cultivation in the culture supernatants of media containing reduced urea concentrations compared to those with higher urea levels (Supplementary Figure S9). However, direct monitoring of intracellular pH throughout the cultivation would be required to confirm this hypothesis. Tdc_*Lb*_, employed here to decarboxylate l-tyrosine to tyramine in strain ATRN, would benefit from a lower intracellular pH, since its reported pH optimum is 5.0, with its activity decreasing by more than 80% at pH values of 7.5 and above [75]. This is consistent with the increased accumulation of l-tyrosine and reduced *N*-acetyltyramine titers observed in the RSM cube portions with elevated urea concentrations, indicating l-tyrosine decarboxylation as a bottleneck for *N*-acetyltyramine production at these media compositions (Supplementary Figure S9).

Evaluation of the optimized medium for the production of additional l-tyrosine derivatives further supports this interpretation (Fig. 8). Tyramine production, which involved only the decarboxylation of l-tyrosine to tyramine, increased by 24% compared to cultivation in standard CGXII medium. In contrast, the production of tyrosol and *N*-acetyltyramine, both requiring additional enzymatic conversion of tyramine, increased by more than 40%, indicating that not only Tdc_*Lb*_ activity but also subsequent pathway enzymes benefit from the optimized conditions. AANAT_*Bm*_, catalyzing the *N*-acetylation of tyramine in strain ATRN, has been described to have a pH optimum of 7.5, with drastically decreased activity at higher pH levels [55]. Similarly, while the pH dependence of the tyramine oxidase from *Kocuria rhizophila* (Tyo_*Kr*_), which is used here for the oxidation of tyramine during tyrosol production, has not yet been characterized, its homolog from *Micrococcus luteus* has a pH optimum around 7, and its activity was shown to decrease at elevated pH values [76]. This indicates that both Tyo_*Kr*_ and AANAT_*Bm*_ might also benefit from the assumed effect of lower urea concentrations on the intracellular pH. Additionally, ammonia is produced during the oxidation of tyramine by Tyo_*Kr*_. Therefore, a lower urea concentration in the culture medium, resulting in a reduced ammonia release during its utilization, may positively affect the thermodynamic equilibrium of tyramine oxidation.

As a second significant medium component, elevated l-phenylalanine concentrations were found to promote *N*-acetyltyramine production, which is in contrast to the original publication describing a 50% reduced l-tyrosine production upon increasing l-phenylalanine supplementation to 1 mM [24]. This effect can be attributed to the synergistic feedback inhibition of the DAHP synthase AroG, which catalyzes the condensation of E4P and PEP as the committed step of the shikimate pathway (Fig. 1), and the chorismate mutase CM, which catalyzes the conversion of chorismate to prephenate as the first branch point in the production of the three aromatic amino acids. While both l-phenylalanine and l-tyrosine independently inhibit AroG and CM activity, the inhibitory effect of the combination of both l-phenylalanine and l-tyrosine is much more pronounced, especially for AroG [77–79]. In this study, however, we observed no significant difference in l-tyrosine production by strain AROM3 in the optimized medium containing 0.65 mM l-phenylalanine compared to the standard medium containing 0.5 mM l-phenylalanine. This may be attributed to combined effects, as the concentrations of not only l-phenylalanine, but also of other factors, especially urea, were adjusted in the optimized medium. The combined effects may balance the negative effect of the increased l-phenylalanine supplementation on l-tyrosine production. This reflects the strength of the DoE approach, which considers the combined effects of different factors rather than optimizing each factor individually. In contrast to l-tyrosine production, the synergistic effect of feedback inhibition by l-phenylalanine and l-tyrosine appears negligible for the production of its derivatives, as the formed l-tyrosine is further converted to tyramine. Particularly during *N*-acetyltyramine production, l-tyrosine concentrations in the culture supernatant remained below 0.1 mM throughout the cultivation (Fig. 7).

Although the optimized medium containing an elevated l-phenylalanine concentration enhanced tyramine, tyrosol, and *N*-acetyltyramine production, l-phenylalanine supplementation at industrial scale would be economically unfavorable due to the associated costs. Therefore, future efforts should focus on genetic engineering strategies to eliminate the need for external l-phenylalanine supply. In strain AROM3, the parental strain of ATRN, exchanging the translational start codon of *pheA* from ATG to TTG resulted in growth deficiency. In contrast, other studies have demonstrated that a start codon exchange to GTG did not cause l-phenylalanine auxotrophy, suggesting that introducing GTG as the start codon for *pheA* expression presents a promising strategy [80]. Alternatively, l-phenylalanine production could be fine-tuned by replacing its native promotor or ribosome binding site to reduce *pheA* expression [81], or by introducing point mutations for a reduced PheA activity [82]. However, since the integration of a gene encoding the phenylalanine-feedback inhibition-resistant *aroG*_*Ec*_^fbr^ [83] in strain AROM3 does not seem to fully compensate for the feedback inhibition of the native AroG, any genetic engineering strategies must be carefully balanced to avoid l-phenylalanine auxotrophy while simultaneously preventing feedback inhibition caused by elevated intracellular l-phenylalanine levels.

The transfer of the *N*-acetyltyramine production by strain ATRN in the optimized medium from cultivation in the BioLector to shake flasks with 10 mL culture medium increased the *N*-acetyltyramine titer by 30%, resulting in a final *N*-acetyltyramine titer of 13.8 ± 0.1 mM (2.5 ± 0.1 g L^− 1^) after 72 h cultivation (Fig. 7). Previously, tyramine production by *E. coli* was demonstrated to benefit from limited dissolved oxygen supply with the authors attributing these effects to reprogramming of metabolic processes promoting tyramine production [84]. Similarly, the reduced oxygen supply during cultivation in shake flasks compared to the BioLector could have benefited *N*-acetyltyramine production here. Compared to the *N*-acetyltyramine production established in this study, a lower titer of 0.85 ± 0.028 g^− 1^ L in minimal medium containing 20 g L^− 1^ glucose has recently been described for an *E. coli* strain engineered for l-tyrosine overproduction and overexpressing genes encoding Tdc_*Lb*_ and AANAT_*Bm*_ [85]. This may be attributed to the fact that *C. glutamicum* has been demonstrated to be more robust than *E. coli* against various aromatic compounds [18], potentially allowing for higher production titers. While the volumetric productivity of 34.4 ± 0.1 mg L^− 1^ h^− 1^ achieved in this study is only less than 10% higher than that of the *E. coli* process (30.5 ± 1.01 mg L^− 1^ h^− 1^), the product yield on substrate was 45% higher with strain ATRN (62.0 ± 0.2 mg g^− 1^ glucose) than with the *E. coli* strain. Notably, unlike the Gram-negative *E. coli*, the Gram-positive *C. glutamicum* does not contain lipopolysaccharides, which can cause pyrogenic responses due to their immunostimulant effect. The absence of lipopolysaccharides facilitates downstream processing and allows for producing GRAS products [86].

Transferring the established *N*-acetyltyramine production to an industrial scale remains a future objective. However, stable upscaling of tyramine production from xylose-based CGXII minimal medium to a 1.5 L stirred bioreactor has previously been demonstrated [23]. Moreover, medium optimization performed in shake flask cultivations for the production of the shikimate pathway-derived PCA by *C. glutamicum* was shown to also enhance PCA production at 5 L scale [34], highlighting the feasibility of reliably upscaling the production of aromatic products by *C. glutamicum* in optimized media. Nevertheless, transferring biotechnological process to an industrial scale may lead to performance losses due to factors such as concentration gradients of feed components or dissolved oxygen [87], genetic stability of the production strain, foam formation, or increased hydrostatic pressure, thereby necessitating extensive process optimization [88].

Cultivating strain ATRN on a mixture of glucose and propionate as carbon sources resulted in the production of 1.6 ± 0.1 mM_Eq*N*-AcTyr_* N*-propionyltyramine, with *N*-propionyltyramine accounting for approx. 34% of the total titer when considering the combined concentrations of l-tyrosine, tyramine, *N*-acetyltyramine, and *N*-propionyltyramine (Fig. 10). These results highlight the potential for future genetic engineering strategies to achieve complete conversion of l-tyrosine and tyramine to *N*-propionyltyramine and to reduce the substrate flux towards *N*-acetyltyramine in favor of *N*-propionyltyramine production. A total yield of 3.7 mM l-tyrosine, tyramine, *N*-acetyltyramine, and *N*-propionyltyramine per mol carbon utilized was achieved on a mixture of glucose and propionate, which was about half of that obtained on glucose as the sole carbon source (6.7 mM l-tyrosine and its derivatives per mol carbon), which might be attributed to the inhibitory effect of propionate. In *Salmonella enterica*, this effect has been shown to result from the accumulation of the propionate catabolic intermediate 2-methylcitrate, which strongly inhibits cell growth [89]. This has been proposed as a possible reason for the reduced growth of *C. glutamicum* on propionate [90]. Moreover, the accumulation of methylmalonyl-CoA and propionyl-CoA leading to a depletion of acetyl-CoA and free CoA has been identified as major factors contributing to the toxic effects of propionate in strains with deleted 2-methylcitrate pathway genes *prpDBC1/2* and cultivated in the presence of vitamin B12 to allow for propionate utilization via the methylmalonyl-CoA pathway, while no depletions of acetyl-CoA or free CoA was observed for the wild-type *C. glutamicum* strain [68]. Overexpression of the propionate catabolism genes might reduce the accumulation of these intermediates, thereby alleviating growth inhibition in *C. glutamicum*, and could simultaneously promote a shift in product formation towards *N*-propionyltyramine. Additionally, since *C. glutamicum* lacks an acetyl-CoA synthetase, overexpression of *E. coli* genes encoding an acetyl-CoA synthetase (*acs*) and a deactylase (*cobB*) might improve acetyl-CoA supply, as it has been demonstrated to increase acetyl-CoA supply for l-leucine production [91]. Moreover, the acetyl-CoA:CoA transferase ActA (cg2840/NCgl2569) has been reported to catalyze the activation of propionate using acetyl-CoA and vice versa [68, 92]. Further research might assess the impact of *actA* deletion on intracellular acetyl-CoA availability, growth, and production on propionate.

Overall, the *de novo N*-propionyltyramine production shown here is a proof-of-concept for broadening the product spectrum of tyramine derivatives by supplementing different acyl-CoA donors. Notably, this is the first study, to our knowledge, describing the biotechnological production of *N*-propionyltyramine. Although no studies have yet investigated the pharmaceutical activities of *N*-propionyltyramine, a structurally related amide isolated from *Tetracentron sinense* bark ((*E*)-3-(4-hydroxyphenyl)-*N*-[2-(4-hydroxyphenyl)-ethyl]prop-2-enamide) has been demonstrated to exhibit activity against a multidrug-resistant leukemia cell line [93]. Furthermore, since *N*-propionyltyramine differs from *N*-acetyltyramine only in the length of the acyl chain, it may display comparable pharmacodynamic activities, such as antimicrobial activity against multidrug-resistant pathogens. At the same time, its longer acyl chain could confer advantageous pharmacokinetic properties. For instance, increased lipophilicity may enhance penetration through biofilms and enhance its uptake into bacterial cells [94]. Moreover, drug permeability across the gastrointestinal membrane and the blood-brain barrier tends to increase with hydrophobicity [95]. Therefore, *N*-propionyltyramine represents a promising candidate for investigation of its pharmaceutical potential.

## Conclusion

We established *de novo* production of *N*-acetyltyramine by overexpression of the gene encoding AANAT_*Bm*_ in a tyramine overproducing *C. glutamicum* strain. Optimization of the plasmid-based expression strength of AANAT_*Bm*_ gene and refinement of the culture medium doubled *N*-acetyltyramine production by strain ATRN, resulting in a final titer of 13.8 ± 0.1 mM (2.5 ± 0.1 g L^− 1^). This highlights the significant impact of combining metabolic engineering strategies with media optimization. Supplementing propionate as a carbon source enabled *N*-propionyltyramine production, demonstrating the potential for expanding the product spectrum of strain ATRN towards promising drug candidates by supplementation of different acyl-donors.

## Supplementary Information

Below is the link to the electronic supplementary material.

Supplementary Material 1

## Data Availability

All data generated or analyzed during this study are included in this published article and its supplementary information files.
