# Supplementary material for: Engineering of Corynebacterium glutamicum for biosynthesis of the pharmaceutically active N-acetyltyramine: establishing and optimizing de novo production
Source: J Biol Eng. 2026 Jun 18;20:105. doi: 10.1186/s13036-026-00713-1 (PMC13281258; doi:10.1186/s13036-026-00713-1)
Supplement: Supplementary file 1 — Supplementary Material 1 [file 13036_2026_713_MOESM1_ESM.pdf]

# Supplementary material to

# Engineering of *Corynebacterium glutamicum* for biosynthesis of the pharmaceutically active *N*-acetyltyramine: Establishing and optimizing *de novo* production

Sara-Sophie Poethe[1], Lilli Sophie Kaimann[1], Kai H. Schülke[2], Stephan C. Hammer[2], Volker F. Wendisch[1*]

[1]Genetics of Prokaryotes, Faculty of Biology and CeBiTec, Bielefeld University, Universitätsstr. 25, 33615 Bielefeld, Germany; sara-sophie.poethe@uni-bielefeld.de (S-S.P.; ORCID 0000-0001-6697-7971); lilli.kaimann@email.uni-freiburg.de (L.S.K.); volker.wendisch@uni-bielefeld.de (V.F.W.; ORCID 0000-0003-3473-0012)

[2]Organic Chemistry and Biocatalysis, Faculty of Chemistry and CeBiTec, Bielefeld University, Universitätsstr. 25, 33615 Bielefeld, Germany; kai.schuelke@uni-bielefeld.de (K.H.S.; ORCID 0000-0003-3486-5400); stephan.hammer@uni-bielefeld.de (S.C.H.; ORCID 0000-0002-3620-9362)

* Correspondence: volker.wendisch@uni-bielefeld.de (V.F.W.); Tel.: +49-521-106-5611; ORCID 0000-0003-3473-0012

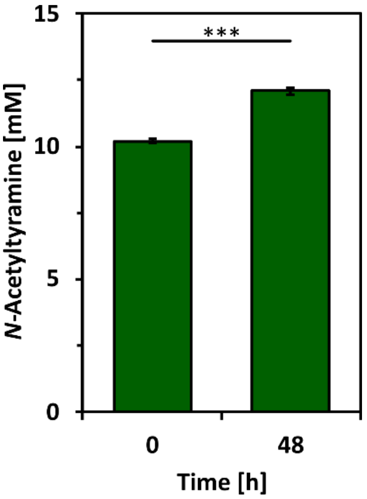


**Supplementary Figure S1 *N*-acetyltyramine degradation test.** Strain AROM3 was cultivated for 72 h in shake flasks in 10 mL CGXII minimal medium containing 40 g $L^{-1}$ glucose, 0.5 mM L-phenylalanine, and 10 mM *N*-acetyltyramine. *N*-Acetyltyramine concentrations were quantified by HPLC at the start and end of cultivation. Values and error bars represent means and standard deviations of triplicate cultivations. Significance was calculated using a two-sided Student's *t*-test with ***: $p < 0.001$.

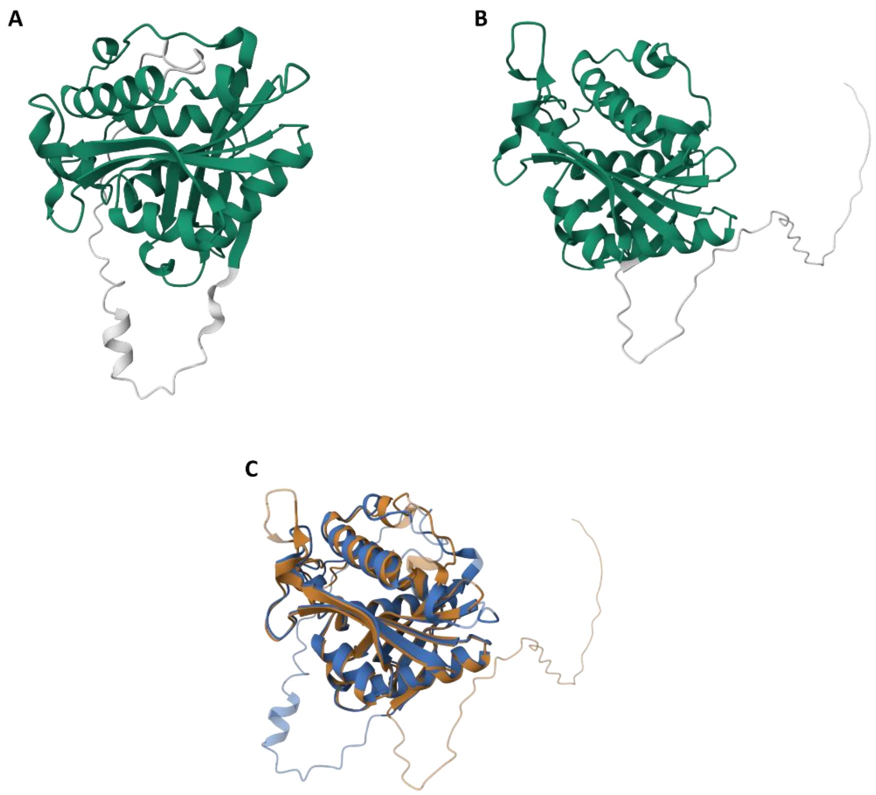


**Supplementary Figure S2 Predicted protein structures and alignments of insect arylalkylamine *N*-acetyltransferases.** 3D structures of $AANAT_{Bm}$ (A) and $AANAT_{Ae}$ (B) were predicted with AlphaFold [1]. The predicted arylalkylamine *N*-acetyltransferase domains of both AANAT proteins, are illustrated in green. A pairwise structure alignment (C) using the RCSB.org alignment tool [2] illustrates the structural similarity between $AANAT_{Bm}$ (blue) and $AANAT_{Ae}$ (orange).

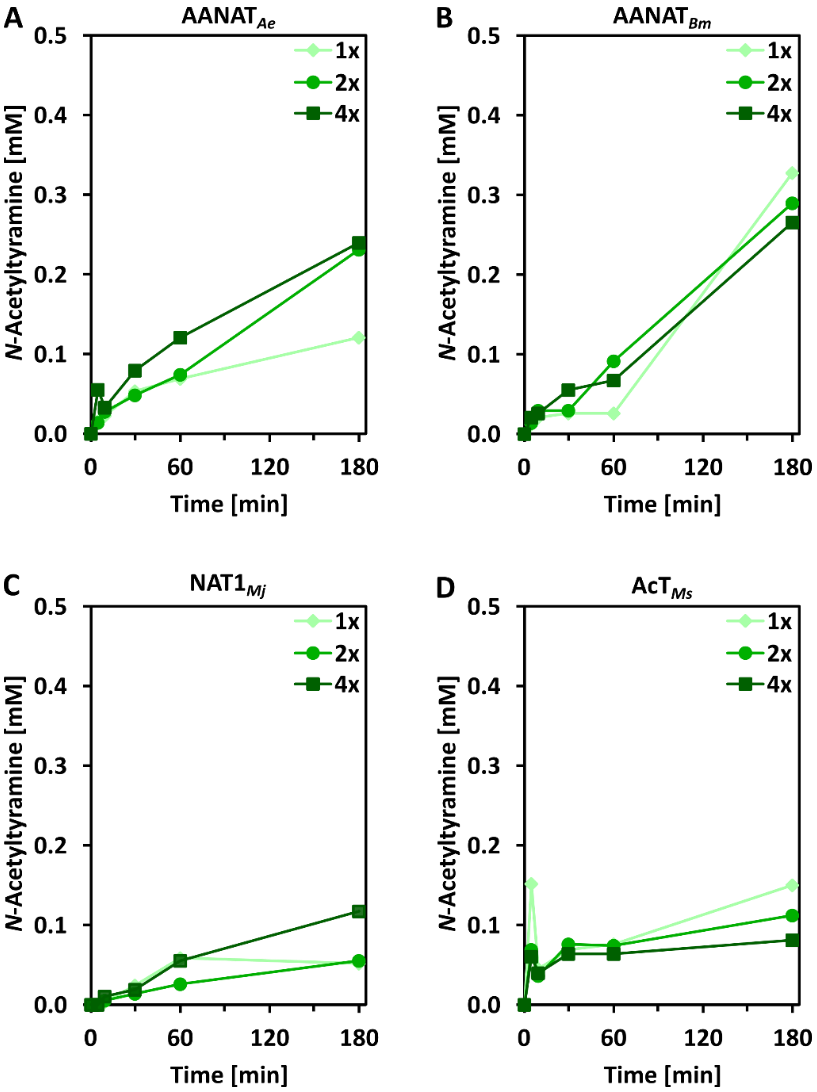


**Supplementary Figure S3 Assaying bacterial and insect enzymes for tyramine acetylation.** Formation of *N*-acetyltyramine within the first 3 h of an *in vitro* assay using a crude extract of *E. coli* DH5α strains carrying plasmids encoding AANAT$_{Ae}$ (A), AANAT$_{Bm}$ (B), NAT1$_{Mj}$ (C), or AcT$_{Ms}$ (D). Darker colors indicate multiples of the crude extract concentrations (1x concentrations of crude extracts were: 0.26 mg mL$^{-1}$ for AANAT$_{Ae}$, 0.17 mg mL$^{-1}$ for AANAT$_{Bm}$, 0.66 mg mL$^{-1}$ for NAT1$_{Mj}$, and 0.78 mg mL$^{-1}$ for AcT$_{Ms}$). No *N*-acetyltyramine was detected for an assay performed with crude extract from an *E. coli* DH5α empty vector control strain.

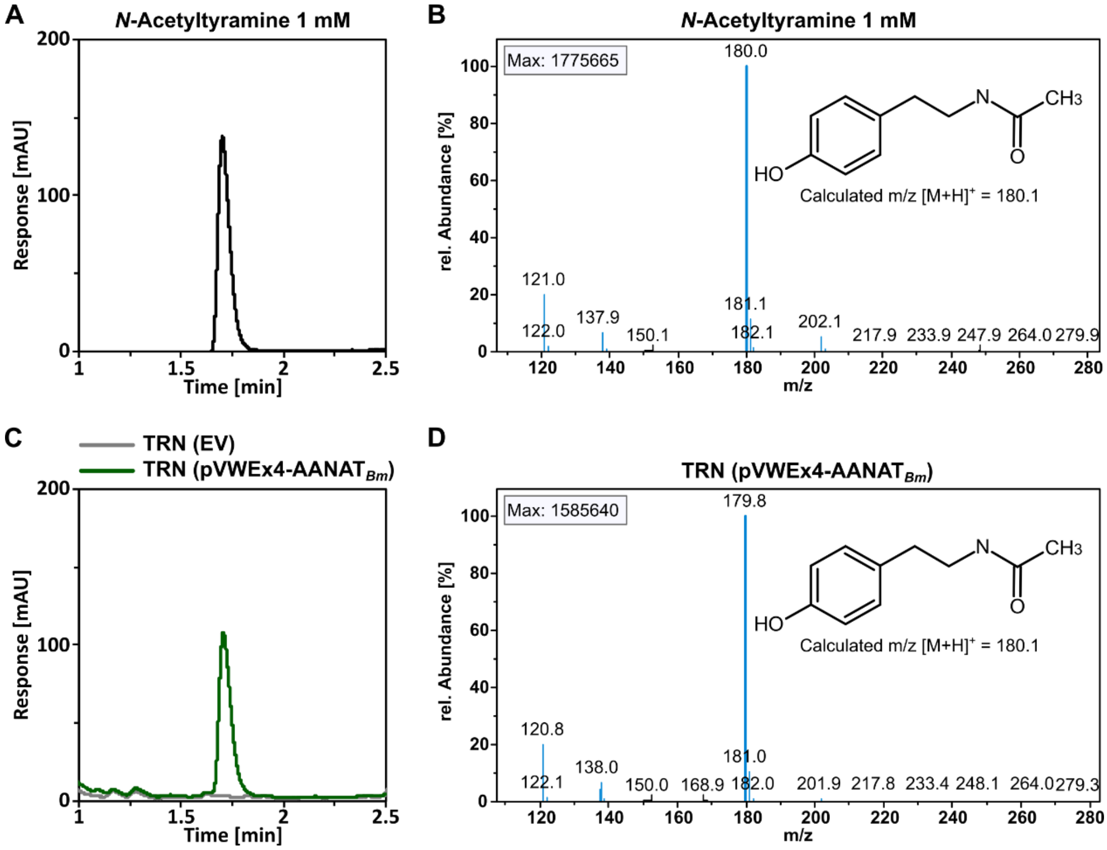


**Supplementary Figure S4 LC/MS analysis of TRN (EV) and TRN (pVWEx4-AANAT$_{Bm}$) culture supernatant.** Compared to the chromatogram of the TRN (EV) supernatant (C, grey), an additional peak at 1.7 min was observed in the DAD chromatogram of the TRN (pVWEx4-AANAT$_{Bm}$) culture supernatant (C, green), recorded at a wavelength of 280 nm, which corresponds to the retention time of the 1 mM *N*-acetyltyramine standard (A). The mass spectrum of the additional peak in the TRN (pVWEx4-AANAT$_{Bm}$) culture supernatant (D) matched that of the *N*-acetyltyramine standard (B), with the most intense signal at m/z 180.1 corresponding to the value of the protonated *N*-acetyltyramine.

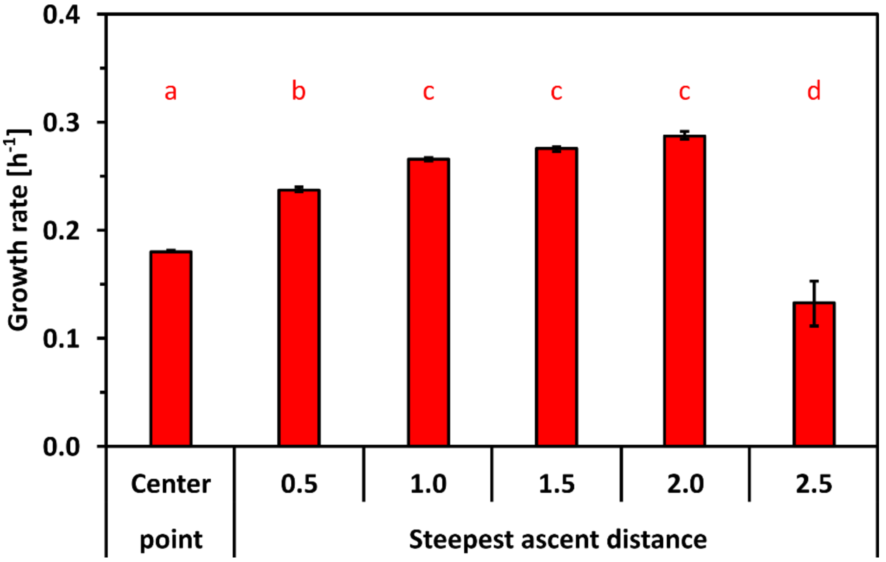


**Supplementary Figure S5 Growth rates of strain ATRN for the medium compositions according to the steepest ascent analysis.** Strain ATRN was cultivated for 120 h in the BioLector cultivation system in 1 mL CGXII minimal medium containing 40 g $L^{-1}$ glucose. The medium composition at the center point corresponds to the standard composition of the CGXII minimal medium. Glucose was depleted at the end of cultivation for all cultivations. Values and error bars represent means and standard deviations from triplicate cultivations. Significance was calculated with an ANOVA followed by a Tukey's honestly significant difference (HSD) test with $\alpha = 0.05$; $p = 2.5e^{-10}$.

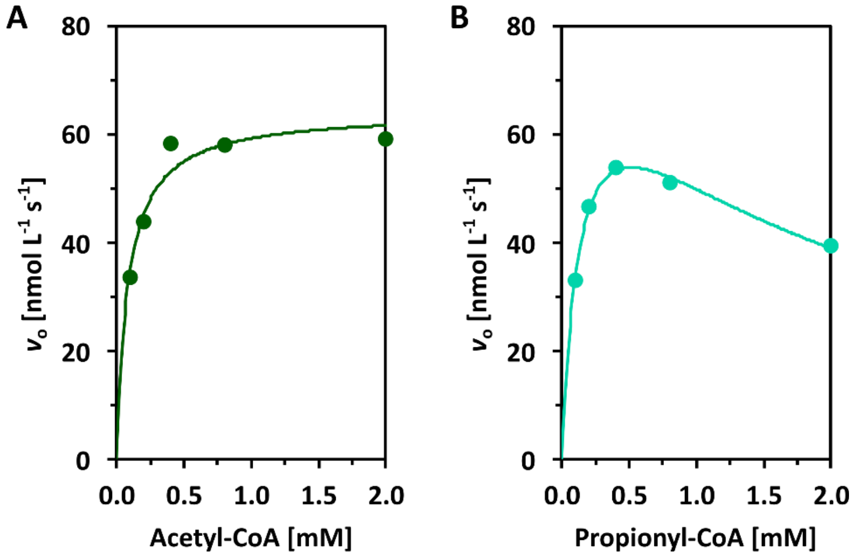


**Supplementary Figure S6 Apparent kinetics of AANAT$_{Bm}$ for acetyl-CoA (A) and propionyl-CoA (B).** AANAT$_{Bm}$ activity was measured in an *in vitro* assay with crude extract from an *E. coli* DH5α strain overproducing AANAT$_{Bm}$. Reactions were performed in 0.1 M phosphate buffer (pH 8.0) containing 10 mM tyramine. Initial velocities $v_o$ measured for the different acyl-CoA concentrations are indicated as points, theoretical curves were calculated with the determined $V_{max}$, $K_M$, and $K_I$ values and are indicated as lines.

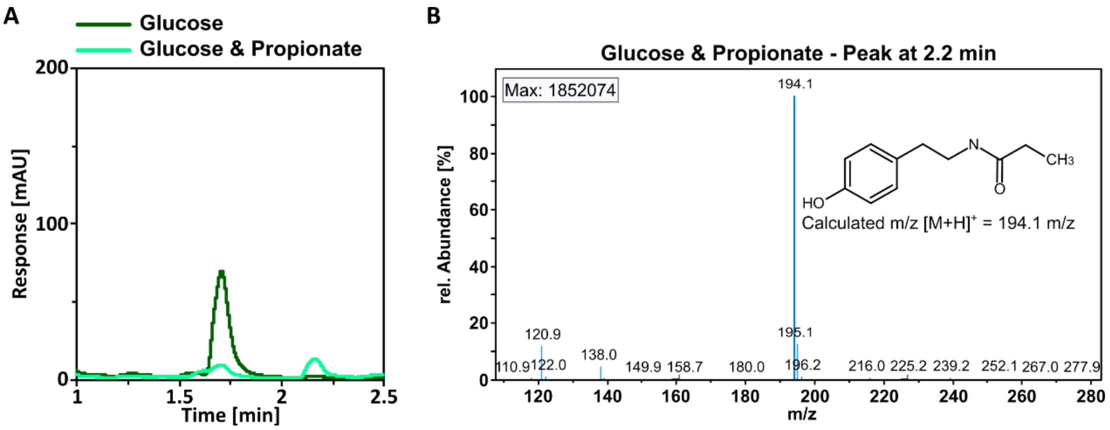


**Supplementary Fig. S7 LC/MS analysis of ATRN culture supernatants after cultivation on a glucose-propionate mixture**. An additional peak was observed at 2.2 min in the DAD chromatogram, recorded at a wavelength of 280 nm, in the supernatant of strain ATRN culture containing a mixture of glucose and propionate (A, light green) compared to strain ATRN cultivated on glucose as sole carbon source (A, dark green). For both culture supernatants, an *N*-acetyltyramine peak was detected after 1.7 min. In the mass spectrum of the additional peak at 2.2 min in the propionate-containing culture supernatant (B), the most intense signal at m/z 194.1 corresponded to the value calculated for protonated *N*-propionyltyramine $[M+H]^+$.

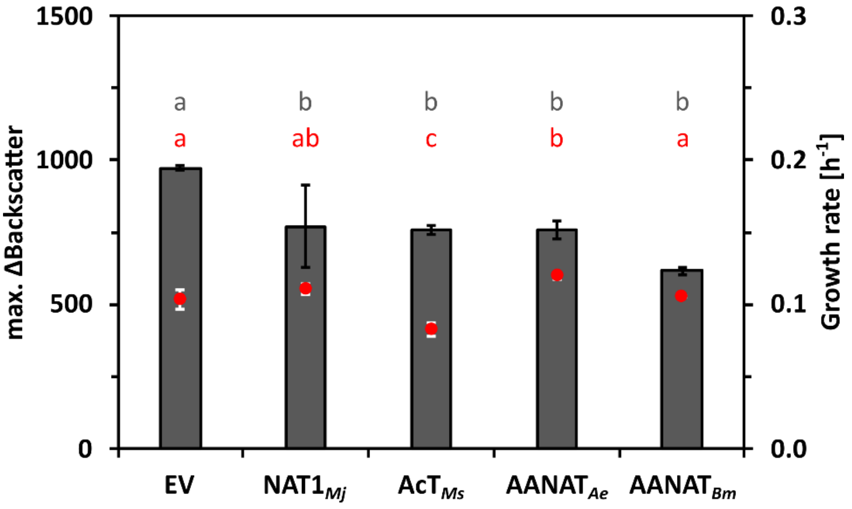


**Figure S8 Growth parameters of strain TRN carrying a pVWEx4-plasmid for the overproduction of different acetyltransferases.** The max. Δbackscatter (grey bars) as a measure of biomass formation and the growth rate (red circles) are indicated. The strains were cultivated for 120 h in the BioLector cultivation system in 1 mL CGXII minimal medium containing 40 g $L^{-1}$ glucose and 0.5 mM L-phenylalanine. Glucose was depleted at the end of cultivation for all cultivations except for strains TRN (pVWEx4-$AcT_{Ms}$) and TRN (pVWEx4-$AANAT_{Bm}$). Values and error bars represent means and standard deviations from triplicate cultivations. Significance was calculated for the max. Δbackscatter (grey letters) and growth rate (red letters) with an ANOVA followed by a Tukey's honestly significant difference (HSD) test with $\alpha = 0.05$; *p* (max. Δbackscatter) = 0.001, *p* (growth rate) = $9.8e^{-6}$.

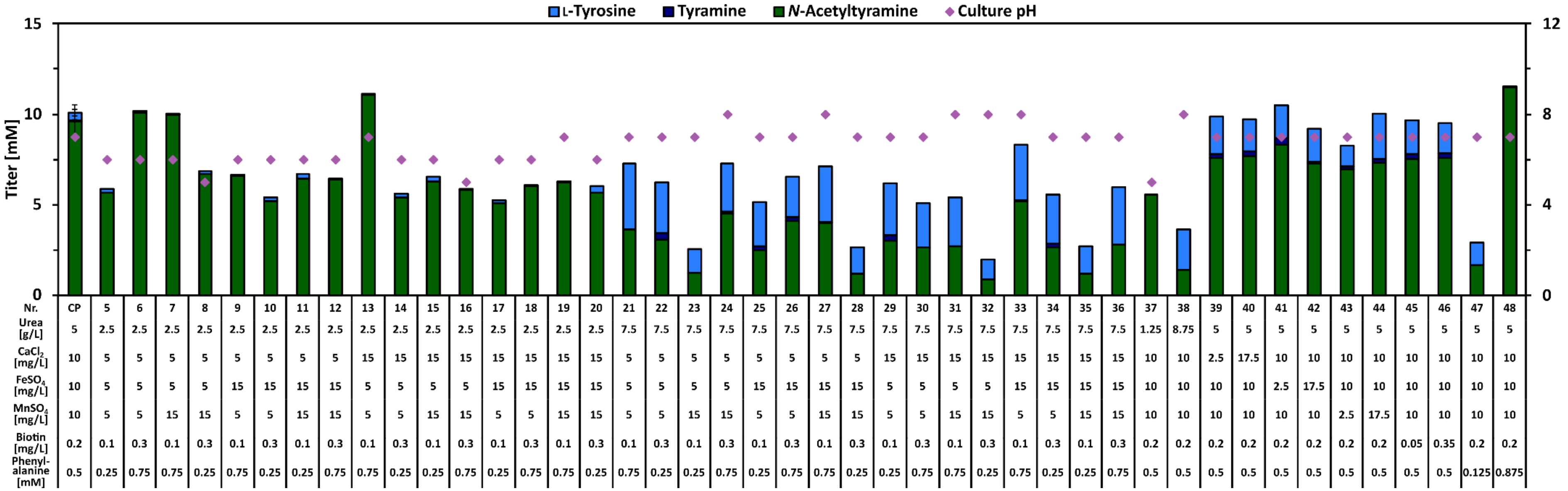


| Nr. | CP | 5 | 6 | 7 | 8 | 9 | 10 | 11 | 12 | 13 | 14 | 15 | 16 | 17 | 18 | 19 | 20 | 21 | 22 | 23 | 24 |
|---|---|---|---|---|---|---|---|---|---|---|---|---|---|---|---|---|---|---|---|---|---|
| Urea [g/L] | 5 | 2.5 | 2.5 | 2.5 | 2.5 | 2.5 | 2.5 | 2.5 | 2.5 | 2.5 | 2.5 | 2.5 | 2.5 | 2.5 | 2.5 | 2.5 | 2.5 | 7.5 | 7.5 | 7.5 | 7.5 |
| $CaCl_2$ [mg/L] | 10 | 5 | 5 | 5 | 5 | 5 | 5 | 5 | 5 | 15 | 15 | 15 | 15 | 15 | 15 | 15 | 15 | 5 | 5 | 5 | 5 |
| $FeSO_4$ [mg/L] | 10 | 5 | 5 | 5 | 5 | 15 | 15 | 15 | 15 | 5 | 5 | 5 | 5 | 15 | 15 | 15 | 15 | 5 | 5 | 5 | 5 |
| $MnSO_4$ [mg/L] | 10 | 5 | 5 | 15 | 15 | 5 | 5 | 15 | 15 | 5 | 5 | 15 | 15 | 5 | 5 | 15 | 15 | 5 | 5 | 15 | 15 |
| Biotin [mg/L] | 0.2 | 0.1 | 0.3 | 0.1 | 0.3 | 0.1 | 0.3 | 0.1 | 0.3 | 0.1 | 0.3 | 0.1 | 0.3 | 0.1 | 0.3 | 0.1 | 0.3 | 0.1 | 0.3 | 0.1 | 0.3 |
| Phenyl-alanine [mM] | 0.5 | 0.25 | 0.75 | 0.75 | 0.25 | 0.75 | 0.25 | 0.25 | 0.75 | 0.75 | 0.25 | 0.25 | 0.75 | 0.25 | 0.75 | 0.75 | 0.25 | 0.75 | 0.25 | 0.25 | 0.75 |

| Nr. | 25 | 26 | 27 | 28 | 29 | 30 | 31 | 32 | 33 | 34 | 35 | 36 | 37 | 38 | 39 | 40 | 41 | 42 | 43 | 44 | 45 | 46 | 47 | 48 |
|---|---|---|---|---|---|---|---|---|---|---|---|---|---|---|---|---|---|---|---|---|---|---|---|---|
| Urea [g/L] | 7.5 | 7.5 | 7.5 | 7.5 | 7.5 | 7.5 | 7.5 | 7.5 | 7.5 | 7.5 | 7.5 | 7.5 | 1.25 | 8.75 | 5 | 5 | 5 | 5 | 5 | 5 | 5 | 5 | 5 | 5 |
| $CaCl_2$ [mg/L] | 5 | 5 | 5 | 5 | 15 | 15 | 15 | 15 | 15 | 15 | 15 | 15 | 10 | 10 | 2.5 | 17.5 | 10 | 10 | 10 | 10 | 10 | 10 | 10 | 10 |
| $FeSO_4$ [mg/L] | 15 | 15 | 15 | 15 | 5 | 5 | 5 | 5 | 15 | 15 | 15 | 15 | 10 | 10 | 10 | 10 | 2.5 | 17.5 | 10 | 10 | 10 | 10 | 10 | 10 |
| $MnSO_4$ [mg/L] | 5 | 5 | 15 | 15 | 5 | 5 | 15 | 15 | 5 | 5 | 15 | 15 | 10 | 10 | 10 | 10 | 10 | 10 | 2.5 | 17.5 | 10 | 10 | 10 | 10 |
| Biotin [mg/L] | 0.1 | 0.3 | 0.1 | 0.3 | 0.1 | 0.3 | 0.1 | 0.3 | 0.1 | 0.3 | 0.1 | 0.3 | 0.2 | 0.2 | 0.2 | 0.2 | 0.2 | 0.2 | 0.2 | 0.2 | 0.05 | 0.35 | 0.2 | 0.2 |
| Phenyl-alanine [mM] | 0.25 | 0.75 | 0.75 | 0.25 | 0.25 | 0.75 | 0.75 | 0.25 | 0.75 | 0.25 | 0.25 | 0.75 | 0.5 | 0.5 | 0.5 | 0.5 | 0.5 | 0.5 | 0.5 | 0.5 | 0.5 | 0.5 | 0.125 | 0.875 |

**Supplementary Figure S9 Titers of L-tyrosine and its derivatives for each condition tested in the RSM.** Titers of L-tyrosine (light blue), tyramine (dark blue), and *N*-acetyltyramine (green), as well as pH values determine in culture supernatants at the end of cultivation using pH test strips (purple diamonds). are indicated. Strain ATRN was cultivated for 120 h in the BioLector cultivation system in 1 mL CGXII minimal medium containing 40 g $L^{-1}$ glucose. The medium composition at the center point corresponds to the standard composition of the CGXII minimal medium. Nr. refers to the media compositions listed in Supplementary Table S3. Values and error bars of the center point represent means and standard deviations of quadruplicate cultivations.

**Supplementary Table S1 Gene sequences used in this work and UniProt Identifier of the encoded protein.**

| Gene name and UniProt ID | Sequence |
|---|---|
| *AANAT$_{Ae}$*<br>F4WZQ5 | CTTAGAAAAATATAATTAATTTTAAAAGGAGGTATTCT**ATG**GAGAATATTCGAGCAAACCGGG<br>TTAACGCTATCCATATTGATAATGACAAAACTATTACTACTAACTTCGTGAAGGATGCACCAAA<br>AATCACTCGCTATTCTCTGGCGGATCTGGCAGACGGTTCATCTAATTCCATGGATTACCACATT<br>GAGATTATCAACAAAGATGATAAGCTTCGCGTGCTTAAGTTTCTCCGCCGCTTTTTTTTTCGC<br>GACGAGCCTCTTAACCAAAATATCCAGCTGATCCCTGAAGGCGAAGATTCCACTTGCGCTGA<br>ACTTGAGGAATATTGCTGTAACGCGAGCTTCGAAAACAATATGTCCCTAATGGCAGTTTCAAC<br>TTCCGGCACCATCGTTGGTGTTCTTCTTAACGGTAAGATGGATAACCTTGTTAGCAACGAAGA<br>ACCAGAATACATTCGATCTTGCAAGAACGCTAAGTTCAAAAAAATTCTGCGCCTCCTTCACTA<br>TCTGGATAAGTCCGTTAATATGGGCGGTCGTTTCCGTGATTCAAAGATCCTTGAGATTCGCATT<br>ATCTCTGTGGACACCAACTGGCGCGGTCGCGGCGTTGGGACTACTCTTATGGGTAAGACCGC<br>TGAAATGGCAAAAGAACAGGGTTACCACTACCTTCGAGCAGACTGCACTTCAATCTTCTCAG<br>CAAAAATGTGTGAACGTCTTGGCTATGATCAAATTTACAAGATCAACTACAAAGACTATGTGG<br>ATGAGGACGGTAAGCCAATTTTCAGCCCTGTTAGCCCACATATTGCTGCAGTTAGCTACGTTA<br>AAAAGCTC**TAA** |
| *AANAT$_{Bm}$*<br>A0EM56 | CGGATAATTTCTACAAAAGGAGGTTTTCT**ATG**GCCGTTACCAGCACCCGCGGCATTGTGAATC<br>TTAAAGAGAAAGGCCATTTTGAGCGAGAAAATGCACGAGTGGAAAAGTACACTCTTGGTGA<br>TGAACTGCCAGCACAGCTATCCTCTCTTGAGATTCGACCTCGTAAGATGAGCGTTCCTGCCTA<br>CACTATCCAGCGACTTACTTACAACGACCGAGATCTAGTTCTGAAGTTCCTTCGCCGCTTCTTC<br>TTTCGTGACGAGCCTATGAATCTTGCAGTTAATCTGCTAGAAACCCCTGAGAGCCGCTGCACT<br>GAGCTTGACGATTATGCAGCGGCCACTCTTTCTGACGGTGTTAGCGTTGCAGCCGTTGACGA<br>GAACGGCGATTACGTTGGTGTTATTATTAACGGAATTGTGCGACGCGAGGAAGTTGATTACA<br>CTGATAAAAGCGAAGATTGCCCTAATCCTAAGTTCCGACGAATTCTGAAGGTTCTGGGACAC<br>CTTGATCGTGAAGCCCGTATTTGGGATAAGCTGCCAGAGACTTGTGATAGCGTTCTTGAGATT<br>CGAATCGCAAGCACCCACTCCAGCTGGCGTGGCCGCGGTCTGATGCGAGTGCTGTGTGAGG<br>AAGCAGAACGACTGGCAAAGGCAATGGGTGCAGGTGCCCTACGCATGGACACCACTAGCGC<br>CTTCAGCGCAGCAGCCGCAGAGCGACTTAATTACAAAATGGCATTTGGAGTTCGATATGCCG<br>ACCTTCCATACGCGCCTCAACCTGAAGCGCCTCACCTAGAAGCACGAGTTTACATTAAAGAG<br>CTG**TAA** |
| *act$_{Ms}$*<br>A0R5U7 | AACGAGACTTACGTAAATATTATATCATAAAAAGGAGGTAATTT**ATG**GCCAAGCGAATTCTGT<br>GTTTCGGTGATTCCCTGACCTGGGGCTGGGTCCCCGTCGAAGACGGGGCACCCACCGAGCG<br>GTTCGCCCCCGACGTGCGCTGGACCGGTGTGCTGGCCCAGCAGCTCGGAGCGGACTTCGA<br>GGTGATCGAGGAGGGACTGAGCGCGCGCACCACCAACATCGACGACCCCACCGATCCGCG<br>GCTCAACGGCGCGAGCTACCTGCCGTCGTGCCTCGCGACGCACCTGCCGCTCGACCTGGTG<br>ATCATCATGCTGGGCACCAACGACACCAAGGCCTACTTCCGGCGCACCCCGCTCGACATCGC<br>GCTGGGCATGTCGGTGCTCGTCACGCAGGTGCTCACCAGCGCGGGCGGCGTCGGCACCAC<br>GTACCCGGCACCCAAGGTGCTGGTGGTCTCGCCGCCACCGCTGGCGCCCATGCCGCACCCCT<br>GGTTCCAGTTGATCTTCGAGGGCGGCGAGCAGAAGACCACTGAGCTCGCCCGCGTGTACAG<br>CGCGCTCGCGTCGTTCATGAAGGTGCCGTTCTTCGACGCGGGTTCGGTGATCAGCACCGAC<br>GGCGTCGACGGAATCCACTTCACCGAGGCCAACAATCGCGATCTCGGGGTGGCCCTCGCGG<br>AACAGGTGCGGAGCCTGCTG**TAA** |
| *NAT1$_{Mj}$*<br>Q98D42 | AGTTACAAGACAGGGAGACCCCTGACCGACCCTAAAAAGGAGGTTTTTG**ATG**AACGACGCG<br>CCCCCTTTCGATCTGGATGCATACCTGGCGCGCATCGGGTATACCGGGCCACGCAACGCATCC<br>CTGGACACTCTGAAGGCACTGCACTTCGCACACCCACAGGCAATCCCTTTCGAAAACATCGA<br>CCCATTTCTAGGGCGCCCAGTGCGTCTGGATCTAGCAGCACTGCAGGATAAGATCGTGCTAG<br>GCGGGCGCGGGGGCTATTGCTTCGAGCACAACCTGCTGTTCATGCACGCACTGAAGGCACT<br>AGGGTTCGAAGTGGGCGGGCTAGCAGCACGAGTGCTGTGGGGCCAGTCCGAAGATGCAAT<br>CACCGCACGCTCCCATATGCTACTACGCGTGGAACTGGATGGCCGTACCTATATCGCAGACGT |

| | |
|---|---|
| | GGGCTTCGGCGGCCTGACCCTGACCGCACCACTGCTGCTGGAGCCAGGCCGCGAGCAGAA AACCCCACATGAACCCTTCCGCATCGTGGAAGCAGATGACCACTTCCGCCTGCAGGCAGCAA TCGGCGGCGATTGGCGCTCCCTGTATCGCTTCGATCTGCAGCCACAATATGAGGTGGACTATT CTGTGACCAATTATTTCCTGTCCACCTCCCCAACATCCCACTTCCTGTCTTCTGTGATCGCAGC GCGCGCAGCACCAGATCGTCGCTATGCACTGCGTGGGAACCGCCTATCCATCCACCATCTAG GCGGCCGCACCGAACAGACAGAAATAGCAACCGCAGCAGATCTAGCAGATACTCTGCAAGG CCTGCTGGGCATCATCATACCTGATCGCACTGCGTTCGAAGCAAAGGTGCGAGAAACTAAGA TCGTGGAAACCAACGCG**TAA** |

Optimized RBS are underlined. Translational start and stop codons are indicated in bold. Genes encoding $AANAT_{Ae}$, $AANAT_{Bm}$, and $NAT1_{Mj}$ were codon-harmonized for *C. glutamicum*. The gene sequence of $act_{Ms}$ is the native sequence.

**Supplementary Table S2 Oligonucleotides used in this work.**

| Purpose | Primer name | Sequence (5' → 3') |
|---|---|---|
| Verification of genes integrated into pSJEx3, pVWEx1, and pVWEx4 | 1143<br>1144 | TTTGCGCCGACATCATAACGGTTCTG<br>CTACGGCGTTTCACTTCTGAGTTCGG |
| Gibson assembly cloning of AANAT$_{Ae}$ gene into pVWEx4 | Fw_aanatAe_pVWEx4<br><br>Rv_aanatAe_pVWEx4 | CCTGCAGGTCGACTCTAGAGCTTAGAAAAATATAAT<br>TAATTTTAAAAGGAGGTATTCTATGGAGAATATTCG<br>CGAGCTCGGTACCCGGGGATCTTAGAGCTTTTTAA<br>CGTAGCTAACTGCAGC |
| Sequencing of AANAT$_{Ae}$ gene | Rv_aanatAe_Seq1<br>Fw_aanatAe_Seq2 | GTTGGTGTCCACAGAGATAATGCG<br>CCTTCACTATCTGGATAAGTCCG |
| Gibson assembly cloning of AANAT$_{Bm}$ gene into pSJEx3, pVWEx1, and pVWEx4 | Fw_aanatBm_pVWEx4<br><br>Rv_aanatBm_pVWEx4 | GCTTGCATGCCTGCAGGTCGACTCTAGAGCGGATA<br>ATTTCTACAAAAGGAGGTTTTCTATGGC<br>CGGCCAGTGAATTCGAGCTCGGTACCCGGGGATCT<br>TACAGCTCTTTAATGTAAACTCGTGCTTCTAGG |
| Sequencing of AANAT$_{Bm}$ gene | Rv_aanatBm_Seq1<br>Fw_aanatBm_Seq2 | GTACCCGGGGATCTTACAGC<br>GGCATTTGGAGTTCGATATG |
| Gibson assembly cloning of AcT$_{Ms}$ gene into pVWEx4 | Fw_actMs_pVWEx4_1<br><br><br>Fw_actMs_pVWEx4_2<br><br>Rv_actMs_pVWEx4 | CGACTCTAGAGAACGAGACTTACGTAAATATTATATC<br>ATAAAAAGGAGGTAATTTATGGCCAAGCGAATTCT<br>GTGTTTCG<br>GCTTGCATGCCTGCAGGTCGACTCTAGAGAACGAG<br>ACTTACGTAAATATTATATC<br>CGAGCTCGGTACCCGGGGATCTTACAGCAGGCTCC<br>GCACC |
| Sequencing of AcT$_{Ms}$ gene | Rv_actMs_Seq1<br>Fw_actMs_Seq2 | CGAAGATCAACTGGAACCAGGGG<br>CGGCACCACGTACCCGGCACCC |
| Gibson assembly cloning of $\mathit{NAT1}_{Mj}$ gene into pVWEx4 | Fw_NAT1Mj_pVWEx4<br><br>Rv_NAT1Mj _pVWEx4 | GCCTGCAGGTCGACTCTAGAGAGTTACAAGACAG<br>GGAGACCCC<br>CGAGCTCGGTACCCGGGGATCTTACGCGTTGGTTT<br>CCACGATCTTAG |
| Sequencing of $\mathit{NAT1}_{Mj}$ gene | Rv_NAT1Mj _Seq1<br>Fw_NAT1Mj_Seq2 | GTGCATAGCGACGATCTGGTGC<br>GACCAATTATTTCCTGTCCACCTCCCC |

**Supplementary Table S3 Setup of the screening design and *N*-acetyltyramine titers obtained for the respective conditions.**

| Nr. | Block | Ammonium sulfate [g $L^{-1}$] | Urea [g $L^{-1}$] | Phosphate [g $L^{-1}$] | MOPS [g $L^{-1}$] | Glucose [g $L^{-1}$] | $CaCl_2$ [mg/L] | $MgSO_4$ x 7 $H_2O$ [mg $L^{-1}$] | $FeSO_4$ x 7 $H_2O$ [mg $L^{-1}$] | $MnSO_4$ x $H_2O$ [mg $L^{-1}$] | $ZnSO_4$ x 7 $H_2O$ [mg $L^{-1}$] | $CuSO_4$ [mg $L^{-1}$] | $NiCl_2$ x 6 $H_2O$ [mg $L^{-1}$] | PCA [mg $L^{-1}$] | Biotin [mg $L^{-1}$] | Phenyl-alanine [mM] | Titer [mM] |
|---|---|---|---|---|---|---|---|---|---|---|---|---|---|---|---|---|---|
| 1 | 1 | 20.0 | 5.00 | 2.0 | 42.0 | 40.0 | 10.0 | 250.0 | 10.0 | 10.0 | 1.00 | 0.20 | 0.020 | 30.0 | 0.20 | 0.500 | 9.0 |
| 2 | 1 | 20.0 | 5.00 | 2.0 | 42.0 | 40.0 | 10.0 | 250.0 | 10.0 | 10.0 | 1.00 | 0.20 | 0.020 | 30.0 | 0.20 | 0.500 | 9.1 |
| 3 | 1 | 20.0 | 5.00 | 2.0 | 42.0 | 40.0 | 10.0 | 250.0 | 10.0 | 10.0 | 1.00 | 0.20 | 0.020 | 30.0 | 0.20 | 0.500 | 8.5 |
| 4 | 1 | 20.0 | 5.00 | 2.0 | 42.0 | 40.0 | 10.0 | 250.0 | 10.0 | 10.0 | 1.00 | 0.20 | 0.020 | 30.0 | 0.20 | 0.500 | 8.0 |
| 5 | 1 | 10.0 | 2.50 | 1.0 | 21.0 | 20.0 | 5.0 | 125.0 | 5.0 | 5.0 | 0.50 | 0.10 | 0.010 | 15.0 | 0.10 | 0.250 | 4.6 |
| 6 | 1 | 10.0 | 2.50 | 1.0 | 21.0 | 60.0 | 5.0 | 125.0 | 5.0 | 5.0 | 1.50 | 0.30 | 0.030 | 45.0 | 0.30 | 0.750 | 8.3 |
| 7 | 1 | 10.0 | 2.50 | 1.0 | 63.0 | 20.0 | 5.0 | 375.0 | 15.0 | 15.0 | 0.50 | 0.10 | 0.010 | 45.0 | 0.30 | 0.750 | 6.1 |
| 8 | 1 | 10.0 | 2.50 | 1.0 | 63.0 | 60.0 | 5.0 | 375.0 | 15.0 | 15.0 | 1.50 | 0.30 | 0.030 | 15.0 | 0.10 | 0.250 | 12.3 |
| 9 | 1 | 10.0 | 2.50 | 3.0 | 21.0 | 20.0 | 15.0 | 125.0 | 15.0 | 15.0 | 0.50 | 0.30 | 0.030 | 15.0 | 0.10 | 0.750 | 4.1 |
| 10 | 1 | 10.0 | 2.50 | 3.0 | 21.0 | 60.0 | 15.0 | 125.0 | 15.0 | 15.0 | 1.50 | 0.10 | 0.010 | 45.0 | 0.30 | 0.250 | 5.0 |
| 11 | 1 | 10.0 | 2.50 | 3.0 | 63.0 | 20.0 | 15.0 | 375.0 | 5.0 | 5.0 | 0.50 | 0.30 | 0.030 | 45.0 | 0.30 | 0.250 | 4.3 |
| 12 | 1 | 10.0 | 2.50 | 3.0 | 63.0 | 60.0 | 15.0 | 375.0 | 5.0 | 5.0 | 1.50 | 0.10 | 0.010 | 15.0 | 0.10 | 0.750 | 11.7 |
| 13 | 1 | 10.0 | 7.50 | 1.0 | 21.0 | 20.0 | 15.0 | 375.0 | 5.0 | 15.0 | 1.50 | 0.10 | 0.030 | 15.0 | 0.30 | 0.250 | 0.0 |
| 14 | 1 | 10.0 | 7.50 | 1.0 | 21.0 | 60.0 | 15.0 | 375.0 | 5.0 | 15.0 | 0.50 | 0.30 | 0.010 | 45.0 | 0.10 | 0.750 | 7.2 |
| 15 | 1 | 10.0 | 7.50 | 1.0 | 63.0 | 20.0 | 15.0 | 125.0 | 15.0 | 5.0 | 1.50 | 0.10 | 0.030 | 45.0 | 0.10 | 0.750 | 1.6 |
| 16 | 1 | 10.0 | 7.50 | 1.0 | 63.0 | 60.0 | 15.0 | 125.0 | 15.0 | 5.0 | 0.50 | 0.30 | 0.010 | 15.0 | 0.30 | 0.250 | 2.3 |
| 17 | 1 | 10.0 | 7.50 | 3.0 | 21.0 | 20.0 | 5.0 | 375.0 | 15.0 | 5.0 | 1.50 | 0.30 | 0.010 | 15.0 | 0.30 | 0.750 | 0.0 |
| 18 | 1 | 10.0 | 7.50 | 3.0 | 21.0 | 60.0 | 5.0 | 375.0 | 15.0 | 5.0 | 0.50 | 0.10 | 0.030 | 45.0 | 0.10 | 0.250 | 6.8 |
| 19 | 1 | 10.0 | 7.50 | 3.0 | 63.0 | 20.0 | 5.0 | 125.0 | 5.0 | 15.0 | 1.50 | 0.30 | 0.010 | 45.0 | 0.10 | 0.250 | 1.2 |
| 20 | 1 | 10.0 | 7.50 | 3.0 | 63.0 | 60.0 | 5.0 | 125.0 | 5.0 | 15.0 | 0.50 | 0.10 | 0.030 | 15.0 | 0.30 | 0.750 | 7.2 |
| 21 | 1 | 30.0 | 2.50 | 1.0 | 21.0 | 20.0 | 15.0 | 375.0 | 15.0 | 5.0 | 1.50 | 0.30 | 0.010 | 45.0 | 0.10 | 0.250 | 3.3 |
| 22 | 1 | 30.0 | 2.50 | 1.0 | 21.0 | 60.0 | 15.0 | 375.0 | 15.0 | 5.0 | 0.50 | 0.10 | 0.030 | 15.0 | 0.30 | 0.750 | 4.8 |
| 23 | 1 | 30.0 | 2.50 | 1.0 | 63.0 | 20.0 | 15.0 | 125.0 | 5.0 | 15.0 | 1.50 | 0.30 | 0.010 | 15.0 | 0.30 | 0.750 | 4.7 |
| 24 | 1 | 30.0 | 2.50 | 1.0 | 63.0 | 60.0 | 15.0 | 125.0 | 5.0 | 15.0 | 0.50 | 0.10 | 0.030 | 45.0 | 0.10 | 0.250 | 8.1 |
| 25 | 1 | 30.0 | 2.50 | 3.0 | 21.0 | 20.0 | 5.0 | 375.0 | 5.0 | 15.0 | 1.50 | 0.10 | 0.030 | 45.0 | 0.10 | 0.750 | 5.4 |
| 26 | 1 | 30.0 | 2.50 | 3.0 | 21.0 | 60.0 | 5.0 | 375.0 | 5.0 | 15.0 | 0.50 | 0.30 | 0.010 | 15.0 | 0.30 | 0.250 | 4.9 |

| | | | | | | | | | | | | | | | | | |
|---|---|---|---|---|---|---|---|---|---|---|---|---|---|---|---|---|---|
| 27 | 1 | 30.0 | 2.50 | 3.0 | 63.0 | 20.0 | 5.0 | 125.0 | 15.0 | 5.0 | 1.50 | 0.10 | 0.030 | 15.0 | 0.30 | 0.250 | 4.7 |
| 28 | 1 | 30.0 | 2.50 | 3.0 | 63.0 | 60.0 | 5.0 | 125.0 | 15.0 | 5.0 | 0.50 | 0.30 | 0.010 | 45.0 | 0.10 | 0.750 | 11.2 |
| 29 | 1 | 30.0 | 7.50 | 1.0 | 21.0 | 20.0 | 5.0 | 125.0 | 15.0 | 15.0 | 0.50 | 0.30 | 0.030 | 45.0 | 0.30 | 0.250 | 0.0 |
| 30 | 1 | 30.0 | 7.50 | 1.0 | 21.0 | 60.0 | 5.0 | 125.0 | 15.0 | 15.0 | 1.50 | 0.10 | 0.010 | 15.0 | 0.10 | 0.750 | 13.2 |
| 31 | 1 | 30.0 | 7.50 | 1.0 | 63.0 | 20.0 | 5.0 | 375.0 | 5.0 | 5.0 | 0.50 | 0.30 | 0.030 | 15.0 | 0.10 | 0.750 | 1.5 |
| 32 | 1 | 30.0 | 7.50 | 1.0 | 63.0 | 60.0 | 5.0 | 375.0 | 5.0 | 5.0 | 1.50 | 0.10 | 0.010 | 45.0 | 0.30 | 0.250 | 2.4 |
| 33 | 1 | 30.0 | 7.50 | 3.0 | 21.0 | 20.0 | 15.0 | 125.0 | 5.0 | 5.0 | 0.50 | 0.10 | 0.010 | 45.0 | 0.30 | 0.750 | 0.0 |
| 34 | 1 | 30.0 | 7.50 | 3.0 | 21.0 | 60.0 | 15.0 | 125.0 | 5.0 | 5.0 | 1.50 | 0.30 | 0.030 | 15.0 | 0.10 | 0.250 | 0.7 |
| 35 | 1 | 30.0 | 7.50 | 3.0 | 63.0 | 20.0 | 15.0 | 375.0 | 15.0 | 15.0 | 0.50 | 0.10 | 0.010 | 15.0 | 0.10 | 0.250 | 1.1 |
| 36 | 1 | 30.0 | 7.50 | 3.0 | 63.0 | 60.0 | 15.0 | 375.0 | 15.0 | 15.0 | 1.50 | 0.30 | 0.030 | 45.0 | 0.30 | 0.750 | 13.8 |
| 37 | 2 | 5.0 | 5.00 | 2.0 | 42.0 | 40.0 | 10.0 | 250.0 | 10.0 | 10.0 | 1.00 | 0.20 | 0.020 | 30.0 | 0.20 | 0.500 | 8.5 |
| 38 | 2 | 35.0 | 5.00 | 2.0 | 42.0 | 40.0 | 10.0 | 250.0 | 10.0 | 10.0 | 1.00 | 0.20 | 0.020 | 30.0 | 0.20 | 0.500 | 5.7 |
| 39 | 2 | 20.0 | 1.25 | 2.0 | 42.0 | 40.0 | 10.0 | 250.0 | 10.0 | 10.0 | 1.00 | 0.20 | 0.020 | 30.0 | 0.20 | 0.500 | 6.4 |
| 40 | 2 | 20.0 | 8.75 | 2.0 | 42.0 | 40.0 | 10.0 | 250.0 | 10.0 | 10.0 | 1.00 | 0.20 | 0.020 | 30.0 | 0.20 | 0.500 | 4.5 |
| 41 | 2 | 20.0 | 5.00 | 0.5 | 42.0 | 40.0 | 10.0 | 250.0 | 10.0 | 10.0 | 1.00 | 0.20 | 0.020 | 30.0 | 0.20 | 0.500 | 0.8 |
| 42 | 2 | 20.0 | 5.00 | 3.5 | 42.0 | 40.0 | 10.0 | 250.0 | 10.0 | 10.0 | 1.00 | 0.20 | 0.020 | 30.0 | 0.20 | 0.500 | 6.6 |
| 43 | 2 | 20.0 | 5.00 | 2.0 | 10.5 | 40.0 | 10.0 | 250.0 | 10.0 | 10.0 | 1.00 | 0.20 | 0.020 | 30.0 | 0.20 | 0.500 | 1.9 |
| 44 | 2 | 20.0 | 5.00 | 2.0 | 73.5 | 40.0 | 10.0 | 250.0 | 10.0 | 10.0 | 1.00 | 0.20 | 0.020 | 30.0 | 0.20 | 0.500 | 7.7 |
| 45 | 2 | 20.0 | 5.00 | 2.0 | 42.0 | 10.0 | 10.0 | 250.0 | 10.0 | 10.0 | 1.00 | 0.20 | 0.020 | 30.0 | 0.20 | 0.500 | 8.5 |
| 46 | 2 | 20.0 | 5.00 | 2.0 | 42.0 | 70.0 | 10.0 | 250.0 | 10.0 | 10.0 | 1.00 | 0.20 | 0.020 | 30.0 | 0.20 | 0.500 | 10.6 |
| 47 | 2 | 20.0 | 5.00 | 2.0 | 42.0 | 40.0 | 2.5 | 250.0 | 10.0 | 10.0 | 1.00 | 0.20 | 0.020 | 30.0 | 0.20 | 0.500 | 6.6 |
| 48 | 2 | 20.0 | 5.00 | 2.0 | 42.0 | 40.0 | 17.5 | 250.0 | 10.0 | 10.0 | 1.00 | 0.20 | 0.020 | 30.0 | 0.20 | 0.500 | 3.5 |
| 49 | 2 | 20.0 | 5.00 | 2.0 | 42.0 | 40.0 | 10.0 | 62.5 | 10.0 | 10.0 | 1.00 | 0.20 | 0.020 | 30.0 | 0.20 | 0.500 | 5.9 |
| 50 | 2 | 20.0 | 5.00 | 2.0 | 42.0 | 40.0 | 10.0 | 437.5 | 10.0 | 10.0 | 1.00 | 0.20 | 0.020 | 30.0 | 0.20 | 0.500 | 6.2 |
| 51 | 2 | 20.0 | 5.00 | 2.0 | 42.0 | 40.0 | 10.0 | 250.0 | 2.5 | 10.0 | 1.00 | 0.20 | 0.020 | 30.0 | 0.20 | 0.500 | 6.0 |
| 52 | 2 | 20.0 | 5.00 | 2.0 | 42.0 | 40.0 | 10.0 | 250.0 | 17.5 | 10.0 | 1.00 | 0.20 | 0.020 | 30.0 | 0.20 | 0.500 | 6.4 |
| 53 | 2 | 20.0 | 5.00 | 2.0 | 42.0 | 40.0 | 10.0 | 250.0 | 10.0 | 2.5 | 1.00 | 0.20 | 0.020 | 30.0 | 0.20 | 0.500 | 6.2 |
| 54 | 2 | 20.0 | 5.00 | 2.0 | 42.0 | 40.0 | 10.0 | 250.0 | 10.0 | 17.5 | 1.00 | 0.20 | 0.020 | 30.0 | 0.20 | 0.500 | 6.1 |
| 55 | 2 | 20.0 | 5.00 | 2.0 | 42.0 | 40.0 | 10.0 | 250.0 | 10.0 | 10.0 | 0.25 | 0.20 | 0.020 | 30.0 | 0.20 | 0.500 | 5.8 |
| 56 | 2 | 20.0 | 5.00 | 2.0 | 42.0 | 40.0 | 10.0 | 250.0 | 10.0 | 10.0 | 1.75 | 0.20 | 0.020 | 30.0 | 0.20 | 0.500 | 5.5 |
| 57 | 2 | 20.0 | 5.00 | 2.0 | 42.0 | 40.0 | 10.0 | 250.0 | 10.0 | 10.0 | 1.00 | 0.05 | 0.020 | 30.0 | 0.20 | 0.500 | 6.9 |

| | | | | | | | | | | | | | | | | | |
|---|---|---|---|---|---|---|---|---|---|---|---|---|---|---|---|---|---|
| 58 | 2 | 20.0 | 5.00 | 2.0 | 42.0 | 40.0 | 10.0 | 250.0 | 10.0 | 10.0 | 1.00 | 0.35 | 0.020 | 30.0 | 0.20 | 0.500 | 6.6 |
| 59 | 2 | 20.0 | 5.00 | 2.0 | 42.0 | 40.0 | 10.0 | 250.0 | 10.0 | 10.0 | 1.00 | 0.20 | 0.005 | 30.0 | 0.20 | 0.500 | 6.8 |
| 60 | 2 | 20.0 | 5.00 | 2.0 | 42.0 | 40.0 | 10.0 | 250.0 | 10.0 | 10.0 | 1.00 | 0.20 | 0.035 | 30.0 | 0.20 | 0.500 | 6.3 |
| 61 | 2 | 20.0 | 5.00 | 2.0 | 42.0 | 40.0 | 10.0 | 250.0 | 10.0 | 10.0 | 1.00 | 0.20 | 0.020 | 7.5 | 0.20 | 0.500 | 1.9 |
| 62 | 2 | 20.0 | 5.00 | 2.0 | 42.0 | 40.0 | 10.0 | 250.0 | 10.0 | 10.0 | 1.00 | 0.20 | 0.020 | 52.5 | 0.20 | 0.500 | 7.6 |
| 63 | 2 | 20.0 | 5.00 | 2.0 | 42.0 | 40.0 | 10.0 | 250.0 | 10.0 | 10.0 | 1.00 | 0.20 | 0.020 | 30.0 | 0.05 | 0.500 | 6.6 |
| 64 | 2 | 20.0 | 5.00 | 2.0 | 42.0 | 40.0 | 10.0 | 250.0 | 10.0 | 10.0 | 1.00 | 0.20 | 0.020 | 30.0 | 0.35 | 0.500 | 6.4 |
| 65 | 2 | 20.0 | 5.00 | 2.0 | 42.0 | 40.0 | 10.0 | 250.0 | 10.0 | 10.0 | 1.00 | 0.20 | 0.020 | 30.0 | 0.20 | 0.125 | 6.4 |
| 66 | 2 | 20.0 | 5.00 | 2.0 | 42.0 | 40.0 | 10.0 | 250.0 | 10.0 | 10.0 | 1.00 | 0.20 | 0.020 | 30.0 | 0.20 | 0.875 | 10.2 |
| 67 | 2 | 20.0 | 5.00 | 2.0 | 42.0 | 40.0 | 10.0 | 250.0 | 10.0 | 10.0 | 1.00 | 0.20 | 0.020 | 30.0 | 0.20 | 0.500 | 6.9 |
| 68 | 2 | 20.0 | 5.00 | 2.0 | 42.0 | 40.0 | 10.0 | 250.0 | 10.0 | 10.0 | 1.00 | 0.20 | 0.020 | 30.0 | 0.20 | 0.500 | 6.3 |
| 69 | 2 | 20.0 | 5.00 | 2.0 | 42.0 | 40.0 | 10.0 | 250.0 | 10.0 | 10.0 | 1.00 | 0.20 | 0.020 | 30.0 | 0.20 | 0.500 | 6.4 |

**Supplementary Table S4 Setup of the RSM design and *N*-acetyltyramine titers obtained for the respective conditions.**

| Nr. | Urea [g $L^{-1}$] | $CaCl_2$ [mg $L^{-1}$] | $FeSO_4$ x 7 $H_2O$ [mg $L^{-1}$] | $MnSO_4$ x $H_2O$ [mg $L^{-1}$] | Biotin [mg $L^{-1}$] | Phenyl-alanine [mM] | Titer [mM] |
|---|---|---|---|---|---|---|---|
| 1 | 5.00 | 10.0 | 10.0 | 10.0 | 0.20 | 0.500 | 10.6 |
| 2 | 5.00 | 10.0 | 10.0 | 10.0 | 0.20 | 0.500 | 10.0 |
| 3 | 5.00 | 10.0 | 10.0 | 10.0 | 0.20 | 0.500 | 9.2 |
| 4 | 5.00 | 10.0 | 10.0 | 10.0 | 0.20 | 0.500 | 8.5 |
| 5 | 2.50 | 5.0 | 5.0 | 5.0 | 0.10 | 0.250 | 5.7 |
| 6 | 2.50 | 5.0 | 5.0 | 5.0 | 0.30 | 0.750 | 10.1 |
| 7 | 2.50 | 5.0 | 5.0 | 15.0 | 0.10 | 0.750 | 10.0 |
| 8 | 2.50 | 5.0 | 5.0 | 15.0 | 0.30 | 0.250 | 6.7 |
| 9 | 2.50 | 5.0 | 15.0 | 5.0 | 0.10 | 0.750 | 6.6 |
| 10 | 2.50 | 5.0 | 15.0 | 5.0 | 0.30 | 0.250 | 5.2 |
| 11 | 2.50 | 5.0 | 15.0 | 15.0 | 0.10 | 0.250 | 6.5 |
| 12 | 2.50 | 5.0 | 15.0 | 15.0 | 0.30 | 0.750 | 6.4 |
| 13 | 2.50 | 15.0 | 5.0 | 5.0 | 0.10 | 0.750 | 11.1 |
| 14 | 2.50 | 15.0 | 5.0 | 5.0 | 0.30 | 0.250 | 5.4 |
| 15 | 2.50 | 15.0 | 5.0 | 15.0 | 0.10 | 0.250 | 6.3 |
| 16 | 2.50 | 15.0 | 5.0 | 15.0 | 0.30 | 0.750 | 5.8 |
| 17 | 2.50 | 15.0 | 15.0 | 5.0 | 0.10 | 0.250 | 5.1 |
| 18 | 2.50 | 15.0 | 15.0 | 5.0 | 0.30 | 0.750 | 6.0 |
| 19 | 2.50 | 15.0 | 15.0 | 15.0 | 0.10 | 0.750 | 6.2 |
| 20 | 2.50 | 15.0 | 15.0 | 15.0 | 0.30 | 0.250 | 5.7 |
| 21 | 7.50 | 5.0 | 5.0 | 5.0 | 0.10 | 0.750 | 3.6 |
| 22 | 7.50 | 5.0 | 5.0 | 5.0 | 0.30 | 0.250 | 3.1 |
| 23 | 7.50 | 5.0 | 5.0 | 15.0 | 0.10 | 0.250 | 1.2 |
| 24 | 7.50 | 5.0 | 5.0 | 15.0 | 0.30 | 0.750 | 4.5 |
| 25 | 7.50 | 5.0 | 15.0 | 5.0 | 0.10 | 0.250 | 2.5 |
| 26 | 7.50 | 5.0 | 15.0 | 5.0 | 0.30 | 0.750 | 4.1 |
| 27 | 7.50 | 5.0 | 15.0 | 15.0 | 0.10 | 0.750 | 4.0 |
| 28 | 7.50 | 5.0 | 15.0 | 15.0 | 0.30 | 0.250 | 1.2 |
| 29 | 7.50 | 15.0 | 5.0 | 5.0 | 0.10 | 0.250 | 3.0 |
| 30 | 7.50 | 15.0 | 5.0 | 5.0 | 0.30 | 0.750 | 2.7 |
| 31 | 7.50 | 15.0 | 5.0 | 15.0 | 0.10 | 0.750 | 2.7 |
| 32 | 7.50 | 15.0 | 5.0 | 15.0 | 0.30 | 0.250 | 0.9 |
| 33 | 7.50 | 15.0 | 15.0 | 5.0 | 0.10 | 0.750 | 5.2 |
| 34 | 7.50 | 15.0 | 15.0 | 5.0 | 0.30 | 0.250 | 2.6 |
| 35 | 7.50 | 15.0 | 15.0 | 15.0 | 0.10 | 0.250 | 1.2 |
| 36 | 7.50 | 15.0 | 15.0 | 15.0 | 0.30 | 0.750 | 2.8 |
| 37 | 1.25 | 10.0 | 10.0 | 10.0 | 0.20 | 0.500 | 5.6 |
| 38 | 8.75 | 10.0 | 10.0 | 10.0 | 0.20 | 0.500 | 1.4 |
| 39 | 5.00 | 2.5 | 10.0 | 10.0 | 0.20 | 0.500 | 7.6 |
| 40 | 5.00 | 17.5 | 10.0 | 10.0 | 0.20 | 0.500 | 7.7 |
| 41 | 5.00 | 10.0 | 2.5 | 10.0 | 0.20 | 0.500 | 8.3 |
| 42 | 5.00 | 10.0 | 17.5 | 10.0 | 0.20 | 0.500 | 7.3 |
| 43 | 5.00 | 10.0 | 10.0 | 2.5 | 0.20 | 0.500 | 7.0 |
| 44 | 5.00 | 10.0 | 10.0 | 17.5 | 0.20 | 0.500 | 7.3 |

| | | | | | | | |
|---|---|---|---|---|---|---|---|
| 45 | 5.00 | 10.0 | 10.0 | 10.0 | 0.05 | 0.500 | 7.5 |
| 46 | 5.00 | 10.0 | 10.0 | 10.0 | 0.35 | 0.500 | 7.6 |
| 47 | 5.00 | 10.0 | 10.0 | 10.0 | 0.20 | 0.125 | 1.6 |
| 48 | 5.00 | 10.0 | 10.0 | 10.0 | 0.20 | 0.875 | 11.5 |

**Supplementary Table S5 Results of the steepest ascent analysis and *N*-acetyltyramine titers obtained for the respective conditions.**

| Distance | Urea [g $L^{-1}$] | $CaCl_2$ [mg $L^{-1}$] | $FeSO_4$ x 7 $H_2O$ [mg $L^{-1}$] | $MnSO_4$ x $H_2O$ [mg $L^{-1}$] | Biotin [mg $L^{-1}$] | Phenyl-alanine [mM] | Titer [mM] |
|---|---|---|---|---|---|---|---|
| 0.0 | 5.00 | 10.0 | 10.0 | 10.0 | 0.20 | 0.50 | 7.4 ± 0.5 |
| 0.5 | 4.26 | 9.5 | 9.0 | 9.7 | 0.19 | 0.58 | 9.7 ± 0.5 |
| 1.0 | 3.92 | 8.8 | 7.2 | 9.4 | 0.18 | 0.65 | 10.7 ± 0.3 |
| 1.5 | 3.69 | 7.9 | 5.0 | 9.3 | 0.17 | 0.70 | 10.5 ± 0.5 |
| 2.0 | 3.50 | 6.8 | 2.8 | 9.2 | 0.16 | 0.75 | 10.1 ± 1.0 |
| 2.5 | 3.32 | 5.7 | 0.6 | 9.2 | 0.16 | 0.78 | 6.4 ± 2.0 |

The concentrations of urea, $CaCl_2$, $FeSO_4$, $MnSO_4$, biotin, and L-phenylalanine were determined performing a steepest ascent analysis based on the previously generated response surface model. Distances up to 2.5 from the center point were tested, since predicted $CaCl_2$- and $FeSO_4$-concentrations became negative for higher distances, which is biologically unfeasible. Obtained *N*-acetyltyramine titers of triplicate cultivations are indicated as mean ± standard deviation.

**Supplementary Table S6 ANOVA analysis results of the screening design predicting the effects on the *N*-acetyltyramine substrate yield.**

| **Factor** | ***t*-value** | **Prob > *t*** |
|---|---|---|
| Intercept | 7.84 | < 0.001 |
| Block | -3.01 | 0.006 |
| Ammonium sulfate | -0.92 | 0.366 |
| Urea | -8.62 | < 0.001 |
| Phosphate | 0.50 | 0.621 |
| MOPS | 4.13 | < 0.001 |
| Glucose | -1.00 | 0.330 |
| $CaCl_2$ | -2.53 | 0.019 |
| $MgSO_4$ | 0.81 | 0.425 |
| $FeSO_4$ | 1.38 | 0.182 |
| $MnSO_4$ | 2.34 | 0.029 |
| $ZnSO_4$ | 0.88 | 0.390 |
| $CuSO_4$ | -0.94 | 0.355 |
| $NiCl_2$ | 0.37 | 0.714 |
| PCA | 1.68 | 0.107 |
| Biotin | -2.44 | 0.023 |
| L-Phenylalanine | 4.30 | < 0.001 |
| Ammonium sulfate$^2$ | -1.17 | 0.254 |
| Urea$^2$ | -1.67 | 0.108 |
| Phosphate$^2$ | -1.83 | 0.081 |
| MOPS$^2$ | 0.66 | 0.518 |
| Glucose$^2$ | -1.58 | 0.127 |
| $CaCl_2{}^2$ | -0.58 | 0.567 |
| $MgSO_4{}^2$ | -0.43 | 0.669 |
| $FeSO_4{}^2$ | -0.45 | 0.660 |
| $MnSO_4{}^2$ | -0.96 | 0.347 |
| $ZnSO_4{}^2$ | 0.12 | 0.908 |
| $CuSO_4{}^2$ | -0.09 | 0.927 |
| $NiCl_2{}^2$ | -1.87 | 0.075 |
| PCA$^2$ | -0.17 | 0.870 |
| Biotin$^2$ | 1.68 | 0.106 |
| L-Phenylalanine$^2$ | -1.17 | 0.254 |
| | **F-value** | **Prob > F** |
| First order | 9.13 | < 0.001 |
| Quadratic effects | 2.32 | 0.036 |
| Lack of fit | 13.08 | 0.005 |

The effect of each factor and its probability, as well as the F-values and probabilities of the model are given. Squared factors indicate quadratic effects. Two-factor interactions were excluded since the strong confounding among the media components did not allow for reliable predictions.

**Supplementary Table S7 ANOVA analysis results of the RSM design predicting the effects on the *N*-acetyltyramine titer.**

| Factor | *t*-value | Prob > *t* |
|---|---|---|
| Intercept | 15.90 | < 0.001 |
| Urea | -7.28 | < 0.001 |
| $CaCl_2$ | -0.90 | 0.377 |
| $FeSO_4$ | -1.36 | 0.189 |
| $MnSO_4$ | -0.98 | 0.338 |
| Biotin | -0.79 | 0.440 |
| L-Phenylalanine | 4.65 | < 0.001 |
| Urea:$CaCl_2$ | 0.25 | 0.802 |
| Urea:$FeSO_4$ | 1.70 | 0.104 |
| Urea:$MnSO_4$ | -0.74 | 0.467 |
| Urea:Biotin | 0.51 | 0.615 |
| Urea:Phe | -0.19 | 0.851 |
| $CaCl_2$:$FeSO_4$ | 0.63 | 0.535 |
| $CaCl_2$:$MnSO_4$ | -1.02 | 0.319 |
| $CaCl_2$:Biotin | -1.13 | 0.272 |
| $CaCl_2$:Phenylalanine | -0.56 | 0.583 |
| $FeSO_4$:$MnSO_4$ | 0.35 | 0.727 |
| $FeSO_4$:Biotin | 0.13 | 0.900 |
| $FeSO_4$:Phenylalanine | -0.75 | 0.459 |
| $MnSO_4$:Biotin | -0.06 | 0.950 |
| $MnSO_4$:Phenylalanine | -0.44 | 0.664 |
| Biotin:Phenylalanine | -0.70 | 0.489 |
| $Urea^2$ | -4.46 | < 0.001 |
| $CaCl_2^2$ | -0.51 | 0.616 |
| $FeSO_4^2$ | -0.36 | 0.720 |
| $MnSO_4^2$ | -0.98 | 0.341 |
| $Biotin^2$ | -0.58 | 0.571 |
| L-$Phenylalanine^2$ | -1.52 | 0.145 |
| | **F-value** | **Prob > F** |
| First order | 13.15 | <0.001 |
| Quadratic effects | 7.84 | <0.001 |
| Lack of fit | 3.21 | 0.183 |

RSM for the effects of urea, $CaCl_2$, $FeSO_4$, $MnSO_4$, biotin, and phenylalanine on the *N*-acetyltyramine titer produced by strain ATRN. The effect of each factor and its probability, as well as the F-values and probabilities of the model are given. A colon between factors indicates two-factor interactions. Squared factors indicate quadratic effects.

## References

1. Jumper J, Evans R, Pritzel A, Green T, Figurnov M, Ronneberger O, et al. Highly accurate protein structure prediction with AlphaFold. Nature. 2021;596:583–9. https://doi.org/10.1038/s41586-021-03819-2.

2. Bittrich S, Segura J, Duarte JM, Burley SK, Rose Y. RCSB protein Data Bank: exploring protein 3D similarities via comprehensive structural alignments. Bioinformatics. 2024;40:btae370. https://doi.org/10.1093/bioinformatics/btae370.
